# Enhanced Control of Single Crystalline Ag Dendritic Growth on Al Foil via Galvanic Displacement and Simultaneous Oxidation of D‐Glucose

**DOI:** 10.1002/smsc.202400478

**Published:** 2025-01-28

**Authors:** Lidija D. Rafailović, Stefan M. Noisternig, Jana Bischoff, Christian Rentenberger, Daniel Bautista – Anguis, Huaping Sheng, Christoph Gammer, Jia Min Chin, Adam Elbataioui, Huanqing Zhang, Jürgen Eckert, Tomislav Lj. Trišović

**Affiliations:** ^1^ Department of Materials Science Chair of Materials Physics Montanuniversität Leoben 8700 Leoben Austria; ^2^ Erich Schmid Institute of Materials Science Austrian Academy of Sciences 8700 Leoben Austria; ^3^ Faculty of Physics Physics of Nanostructured Materials University of Vienna 1090 Vienna Austria; ^4^ Polymer Competence Center Leoben GmbH 8700 Leoben Austria; ^5^ Institute of Inorganic Chemistry – Functional Materials Faculty of Chemistry University of Vienna 1090 Vienna Austria; ^6^ Institute of Technical Sciences of the Serbian Academy of Sciences and Arts Belgrade 11000 Serbia

**Keywords:** Ag single crystalline dendrites, D‐gluconic acids, electrospinning, galvanic displacements, polyacrylonitrile nanofiber templates

## Abstract

A facile synthesis platform for the formation of stable single crystalline Ag dendrites is demonstrated. Using a porous electrospun polyacrylonitrile nanofiber network on Al foil as a template facilitates more uniform dendritic growth in the presence of D‐glucose. In contrast, a denser polymer network restricts the nucleation site availability on the Al foil, highlighting the critical role of the substrate. The growth formation of silver dendrites is reduced in the solution when two simultaneous processes occur: The electroreduction of Ag^+^ in the D‐glucose solution and galvanic displacement driven by the interaction of Ag^+^ with the aluminum substrate. High‐resolution transmission electron microscopy analysis shows the single crystalline nature of Ag dendrites grown from the Al substrate, revealing atomic structures with closely packed layers forming highly faulted face‐centered cubic and hexagonal close‐packed structures. The remarkable long‐term stability of Ag dendrites is primarily attributed to their single crystalline structure, with additional contributions from capping by D‐gluconic acid, as confirmed by Raman analysis. This novel approach to the generation of highly stable Ag dendrites has significant potential for applications such as surface‐enhanced Raman scattering, which has to date been considered to be very sensitive to environmental effects.

## Graphical abstract

The study demonstrates controllable growth of single crystalline Ag dendrites via galvanic displacement on Al foil, accompanied by concurrent D‐glucose oxidation in the solution. Porous electrospun polymer networks serve as template, limiting Ag^+^ access to the foil. The stability of Ag dendrites is attributed to their single crystalline structure and capping by D‐gluconic acid, a product of D‐glucose oxidation.

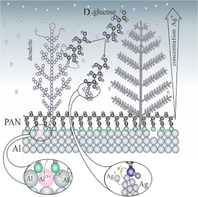

## 1 Introduction

Electrospinning is a nanofabrication method finding widespread use in many technologically relevant fields.^[^ ^1^, ^2^, ^3^, ^4^ ^]^ Its versatility, ease of processing, and the ability to tailor the composition of electrospun polymer nanofibers (NFs) to yield composites with desirable properties such as high surface areas and microporosity are particularly advantageous for applications such as catalysis and biosensing.^[^ ^5^, ^6^ ^]^ Numerous approaches have been reported in the literature, including in situ core–shell or nanoparticle fiber synthesis, or surface functionalization of electrospun nanofiber mats.^[^ ^1^, ^7^, ^8^, ^9^ ^]^

Among the polymers used for electrospinning, polyacrylonitrile (PAN) stands out for its chemical stability and its ability to yield carbon nanofibers for high‐end applications after thermal treatment.^[^ ^10^, ^11^, ^12^, ^13^ ^]^

Silver, first reported to exhibit surface‐enhanced Raman scattering (SERS) effect by Fleishmann et al. on roughened surfaces,^[^ ^14^ ^]^ continues to attract the attention of researchers.^[^ ^15^ ^]^ In particular, Ag nanostructures, fabricated in the form of nanoparticles, nanowires, nanorods, or dendrites, were reported to enhance the optical, plasmonic, or catalytic properties of nanomaterials.^[^ ^16^ ^]^ Dendritic Ag structures enhance weak Raman signals through their corrugated surfaces, primarily through electromagnetic enhancement based on local surface plasmon resonance and, to a lesser extent, through chemical enhancement by analyte binding.^[^ ^17^ ^]^ Dendritic Ag having atoms at abundant sharp corners and edges is considered one of the most effective SERS templates for rapid and nondestructive detection of a variety of probe molecules.^[^ ^9^, ^18^, ^19^ ^]^ Still, long‐term stability and applications of Ag structures with corrugated surfaces containing a high density of steps, and kinks, are impeded due to the lower energy barrier for atomic rearrangements enhancing the mobility of silver and undesired chemical interactions. Therefore, strategies to fabricate highly sensitive and stable SERS substrates to enhance the durability of fabricated Ag dendrites remain a challenge and are the focus of recent studies.^[^ ^17^, ^20^ ^]^

Many synthesis methods of dendritic Ag materials have been reported, including microwave,^[^ ^21^ ^]^ photochemical,^[^ ^6^ ^]^ sonochemical,^[^ ^22^ ^]^ or electrochemical routes.^[^ ^17^, ^23^ ^]^ Among these, electroless deposition is an attractive platform due to its scalability and fast processing. In wet synthesis methods, metal atoms in the solution form through electrons supplied by a reducing agent, leading to a series of nucleation and crystallization steps that result in metal nanostructures.^[^ ^24^ ^]^ The process of “electroless deposition” relies on three different mechanisms: autocatalysis, substrate‐assisted catalysis, and galvanic displacement (GD).^[^ ^25^ ^]^ In recent years, the GD reaction, driven by a favorable difference between reduction potentials between metal pairs,^[^ ^25^ ^]^ has emerged as an effective approach to synthesize various nanostructures of noble metals (such as Au, Ag, Pd, and Pt) and their alloys. GD uniquely yields Ag structures via displacement with metals such as Cu, Fe, Zn, Mg, or Sn which bear favorable standard electrode potential differences with Ag.^[^ ^16^ ^]^ The spontaneous silver reduction by GD is even more favored when paired with aluminum, due to a large standard electrode potential difference between Al^3+^/Al (−1.663 V versus standard hydrogen electrode (SHE)) and Ag^+^/Ag (+0.799 V versus SHE). However, in practice, the native oxide layer on Al suppresses galvanic coupling and often hazardous etchants as HF or additional metals as Cu are necessary to trigger the reaction and displace Al.^[^ ^26^ ^]^ Therefore, the preparation of such SERS substrates requires multiple processing steps, including Al substrate etching and activation in several solutions.^[^ ^19^ ^]^ Furthermore, controlling Ag growth is challenging without using metallic or nonmetallic templates as anodic aluminum oxide (AAO). Our previous results suggest that such steps can be avoided by electrochemical Al oxidation during Ag dendrite deposition, which simultaneously functionalizes AAO pores.^[^ ^27^ ^]^ The addition of surfactants can induce Ag growth in specific directions or lead to the formation of single crystalline silver structures.^[^ ^16^ ^]^ Besides, Ag complexation in solution can reduce the standard electrode potential needed to drive the GD reaction.^[^ ^28^ ^]^ However, although the addition of surfactants or weak reducing agents can enhance the synthesis process, they often limit its applicability by negatively affecting the enhancement of the weak Raman signal for the detection of target analytes.

Our study demonstrates enhanced control in the growth of Ag dendrites on Al foil driven by the GD reaction in the presence of added D‐glucose to a Tollens‐based reagent. This process relies on the interplay between the GD reaction, involving Ag^+^ in the solution and Al foil, and a competitive autocatalytic electroless reduction process occurring simultaneously in the solution. The resulting Ag structures are well anchored to the Al foil and located beneath the PAN polymer fiber mats, which serves as a porous template to mediate dendritic growth. High‐resolution transmission electron microscopy (HRTEM) analysis confirms a single crystalline structure of the fabricated Ag dendrites, exhibiting highly faulted face‐centered cubic (FCC) and hexagonal close‐packed (HCP) structures. Scanning electron microscopy (SEM) analysis conducted after 1 year of storage under ambient conditions highlights their remarkable long‐term stability. This stability is primarily attributed to the single crystalline structure, which minimizes grain boundaries, reducing reactivity and susceptibility to oxidation. Furthermore, we assume that the stability is enhanced by D‐gluconic acid, acting as a capping agent, formed during the oxidation of D‐glucose occurring simultaneously in the solution.

Our approach opens a new avenue for preparing SERS template platforms using simple and green organic reagents such as sugars to control the Ag dendritic growth. Moreover, such a template‐mediated approach further exploring surface treatment and patterning of inexpressive Al foil could be used for selective deposition.

## 2 Results and Discussions

### 2.1 PAN NF Formation

PAN nanofiber mats were electrospun onto Al foil supports. To verify the formation of PAN fibers, Fourier‐transform infrared spectroscopy (FTIR) analysis was carried out on the fiber mats collected on the Al foil. The characteristic band of polyacrylonitrile at 2244 cm^−1^ found in the acquired FTIR spectrum is assigned to the stretching vibration of C ≡ N, indicating the successful fabrication of the mats comprised of PAN.^[^ ^10^, ^29^ ^]^ The bands at 2922 and 1453 cm^−1^ are attributed to the C—H stretching and C—H bending of PAN, respectively. The weak intensity of the peak at 1254 cm^−1^ is assigned to C—N stretching in the backbone PAN structure,^[^ ^30^ ^]^ while the peak at 1670 cm^−1^ is assigned to the vibration mode of the carbonyl group due to residual dimethyl formamide (DMF) solvent from the PAN electrospinning solution. The broadband in the region from 3300 to 3700 cm^−1^ is indicative of O—H stretching vibrations from the absorbed water.

**Figure 1:**
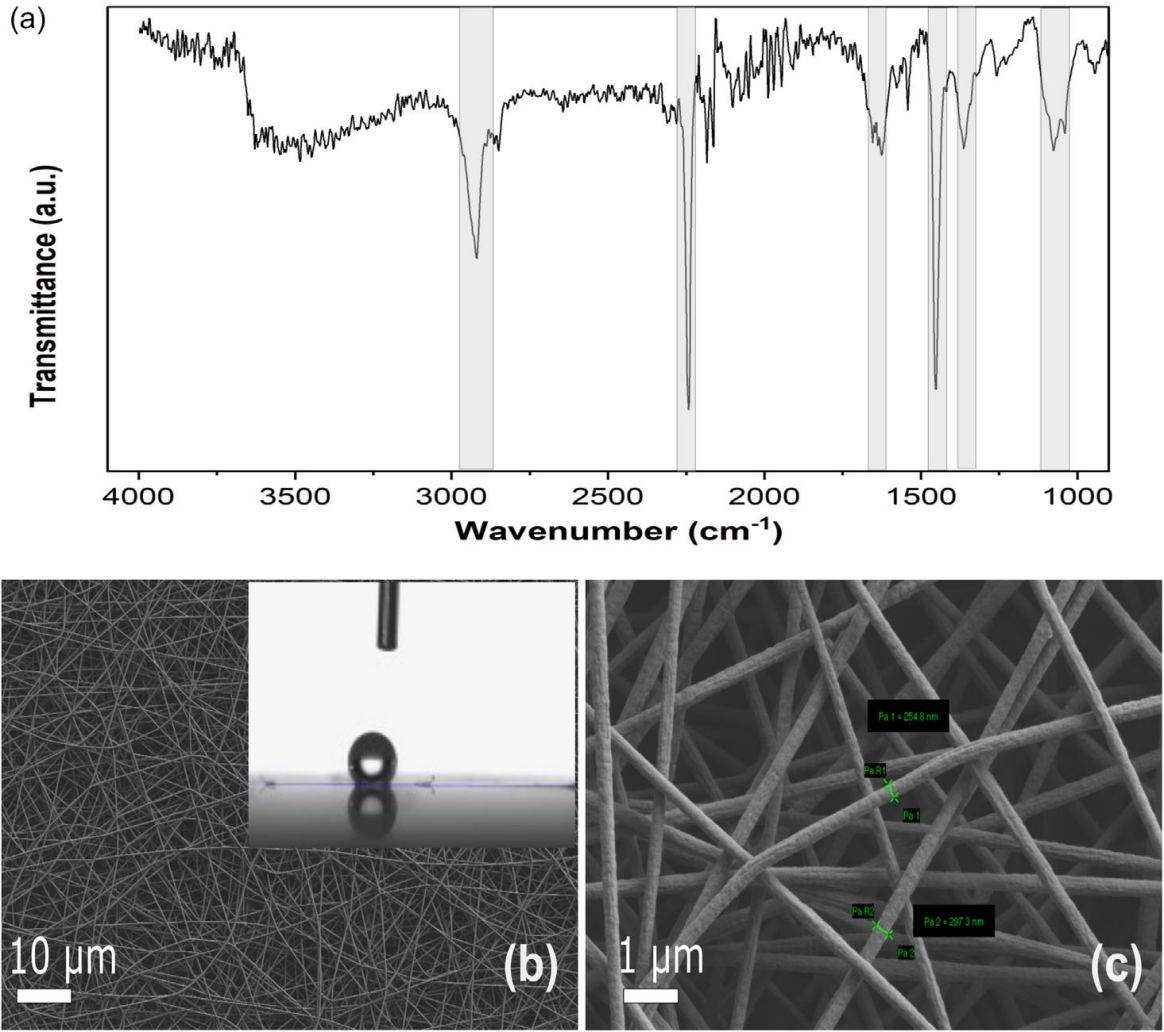
a) A typical FTIR spectrum of electrospun PAN NF mats on Al foil and b) SEM image of electrospun PAN NFs showing in the inset hydrophobicity, as determined by the measured water contact angle of 120°. c) A detailed examination of the surface topology of PAN NFs showing an average fiber diameter size of around 250 nm.

The hydrophobic nature of the PAN NF mats is determined by the measured water contact angle of around 120°, suggesting a low wettability of the PAN NF (inset **Figure** **1**b) with an average fiber diameter size of around 250 nm, as depicted in Figure 1c.

### 2.2 Surface Functionalization with Ag Structures: Dependence on the Support and Reaction Selection

**Figure 2:**
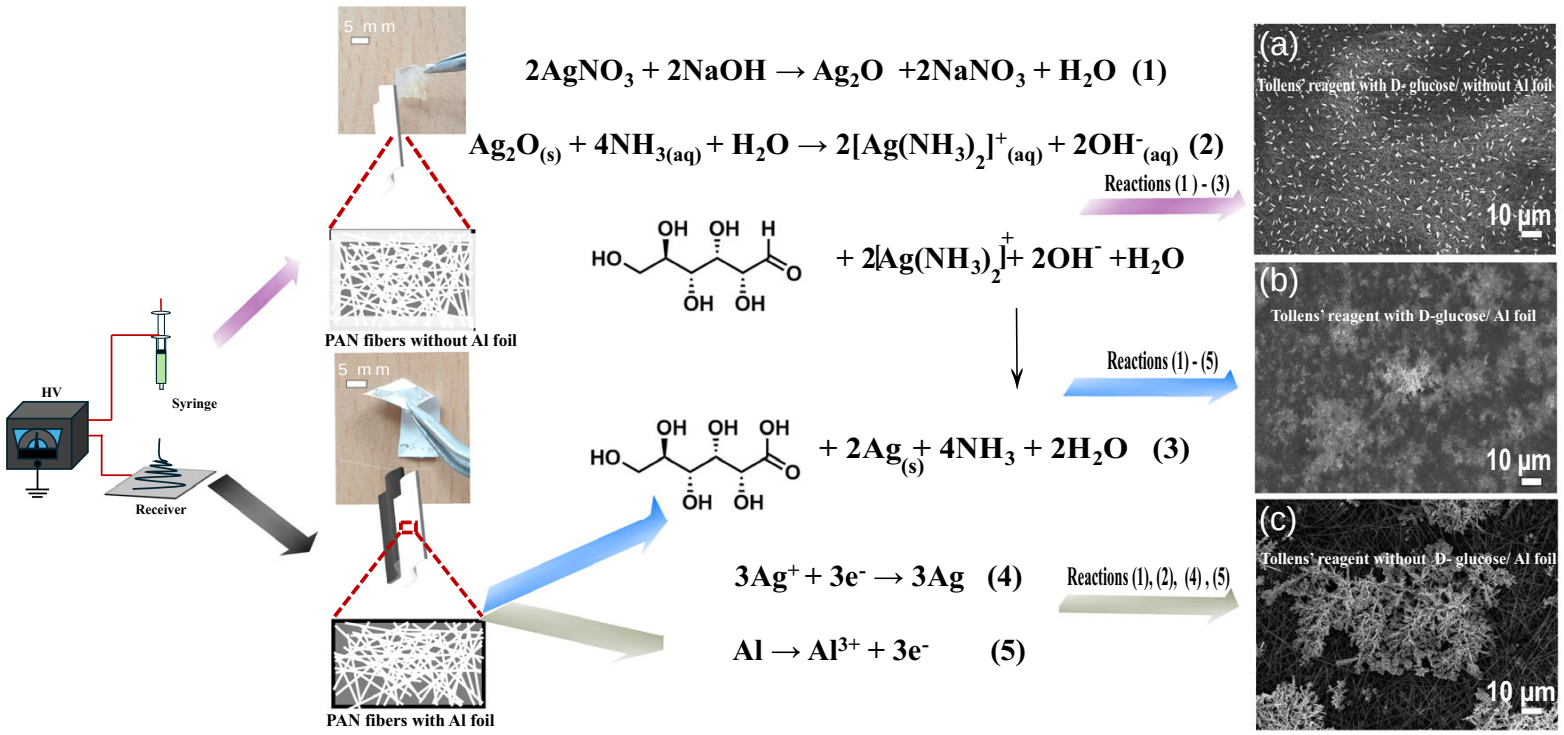
Schematic description of different Ag structures formed during the reaction time of *t* = 180 s (depicted in acquired SEM images), divided in dependence of PAN NF supports and reactions in the solution: a) Ag platelet formation on PAN/filter paper support in Tollens’ regent; b) Ag single crystalline dendrite formation triggered by the Al foil, in the concurrent reaction between GD and Ag autocatalytic reaction undergoing Tollens’ reaction; and c) massive Ag dendrite formation mediated by the GD on the Al foil in the absence of D‐glucose in Tollens’ reagent.

The Ag morphology and structure depend on the selection of the electrospun support and the presence of D‐glucose in Tollens’ reagent, which allows tailoring of the silver structure through time, concentration, or temperature conditions.^[^ ^31^ ^]^ Typically, synthesis of Ag dendritic structures was carried out by immersing several cm^2^ of Al foil supported by PAN NF mat in Tollens’ reagent. By the Tollens’ reaction, the reduction of Ag^+^ ions to elemental Ag is achieved using D‐glucose as reducing agent in an alkaline solution, following Reaction (1)–(3), shown in **Figure** **2**. The galvanic exchange on the Al foil is described by Reaction (4) and (5), as illustrated in Figure 2.

To study the synergy and the influence of GD reaction on the formation of Ag dendrites, we carried out the following experiments: 1) in the absence of Al foil supports and 2) in the absence of D‐glucose in the solution. All other conditions were kept the same, that is, identical concentration of AgNO_3_ and NaOH and the same volume of added NH_3_ to redissolve Ag_2_O oxide precipitates (Reaction (1)) to Ag complexes (Reaction (2)), including the same geometrical surface area of electrospun PAN nanofibers and the same reaction time *t* = 180 s.

We hypothesize that an electrical field driven by a galvanic coupling reaction is an essential factor to synthesize finely branched Ag dendrites and investigated this by replacing the Al foil with a nonmetallic substrate and varying the experimental conditions: 1) The absence of metallic Al foil (Figure 2a and S1a,b, Supporting Information) supports the synthesis path with the GD reaction as the main driving force for dendritic Ag growth. SEM images reveal that only Ag platelets were formed in the absence of the Al foil when the electrospun PAN NFs were fabricated on filter paper (under the same conditions as for electrospinning on Al foil). 2) The absence of glucose as an external reducing agent (Figure 2c and S1c,d, Supporting Information) results in the formation of larger but rougher branched dendrites. In contrast to case (1), a higher Ag^+^ concentration is available since it has not been consumed by the concurrent glucose reaction. This leads to mixed Ag deposition in the form of (d.1) large dendrites and (d.2) attached particles as showin in Figure S1, Supporting Information. Accordingly, SEM images in the absence of glucose also clearly indicate that dendritic Ag growth is governed by galvanic coupling of the redox pair.

To summarize, distinct Ag structures emerge: using a nonmetallic substrate results in Ag platelet formation following the Reaction (1)–(3) (Figure 2a); finely branched Ag dendrites grow through the PAN fiber porous network on the Al foil according to Reaction (1)–(5) (Figure 2b); a mixture of Ag particles and branched Ag structures (Figure 2c) forms without D‐glucose, solely relying on the GD reaction as a method for Ag functionalization.

**Figure 3:**
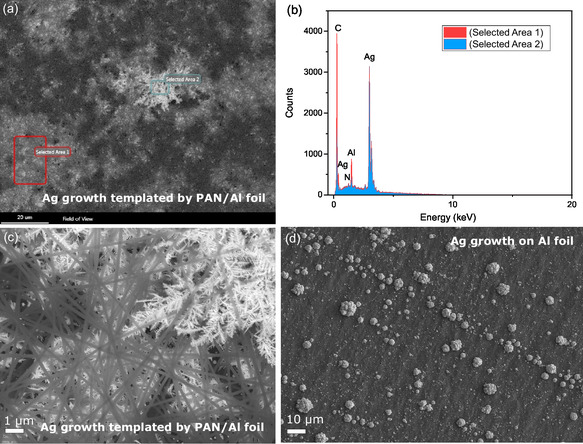
a) SEM image showing finely branched Ag dendritic structures mediated by PAN NFs electrospun on an Al foil template upon immersion into Tollens’ reagent. b) EDX spectra of selected area 1 representing the Ag dendrites surrounded by PAN NFs and selected area 2 representing Ag branches exposed to the top surface, c) SEM image showing a more detailed insight into the branched Ag dendrites grown from the Al foil used as support below the visible porous PAN NFs, and d) SEM image of the bare Al foil immersed into Tollens’ reagent, showing that Ag deposition occurs initially after 30 s as nanoparticles grow into big agglomerates on the Al foil without PAN fibers.

The evolution of the morphological complexity of the fabricated silver structures was monitored by varying the reaction time and Ag^+^ concentration in the solution. A template‐mediated growth of Ag dendrites was accomplished using a porous electrospun PAN NF network on an Al foil by immersion into Tollens’ reagent for 180 s (**Figure** **3**).

SEM images depict the resulting structure of finely branched Ag dendrites on the Al foil visible through the porous PAN network (Figure 3a,c). Corresponding energy‐dispersive X‐ray spectroscopy (EDX) of the Ag dendrites (Figure 3b) reveals that 2 randomly selected dendritic Ag areas possess the same elements of C, Al, and Ag, differing only in their relative amounts, which we attribute to the different density of the PAN NFs and the Ag dendrites. In contrast to very finely branched Ag dendrites grown from the Al foil through the fiber network (Figure 3c), the bare Al foil immersed in Tollens’ reagent shows a nonuniform initial deposition of Ag in the form of NPs and larger agglomerates (Figure 3d).

To elucidate the main mechanism of Ag dendrite formation, we carry out synthesis on previously surface‐treated Al foil. Figure S2, Supporting Information, illustrates the differences in Ag growth on such Al foils without PAN porous network after immersion in the Tollens‐based reagent for the same duration. Large, well‐developed dendrites are observed (Figure S2a,b, Supporting Information) on surface‐treated Al foil showcasing the growth driven by the GD. The addition of the D‐glucose results in only partial particle coverage and localized Ag growth evident at specific sites of Al foil (Figure S2c,d, Supporting Information). These findings highlight that Ag nucleation is highly dependent on the Al foil and its surface pre‐treatment.

#### 2.2.1 Formation of Dendritic Structures Templated by the Porosity of the Electrospun Nanofiber Network

**Figure 4:**
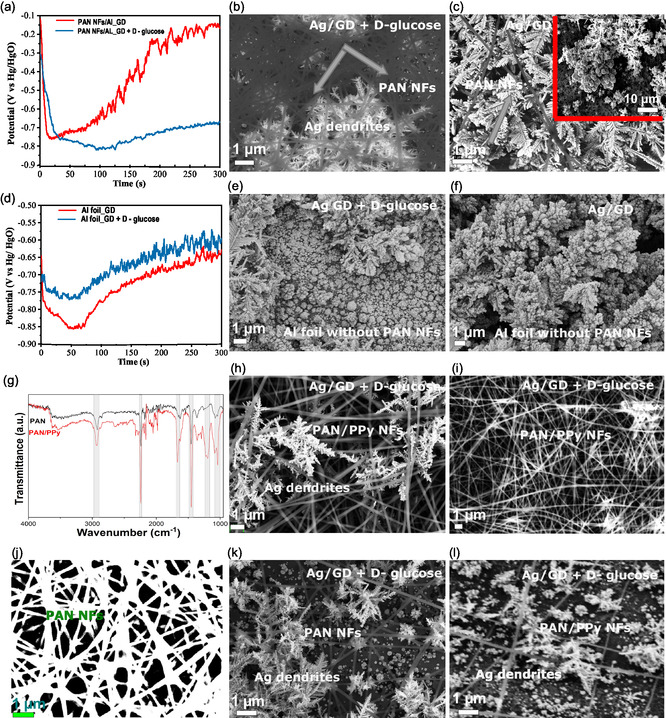
Influence of the synergy between D‐glucose and GD reaction on Ag growth by monitoring: a) OCP of electrospun NFs on Al foil and SEM images of b) finely branched Ag dendrites grown through the PAN NFs network and c) massive Ag dendrite structures grown in the absence of D‐glucose. Comparison to blank Al foil made by monitoring of d) OCP and SEM images showing growth of Ag dendrites and particles in the e) presence and f) absence of D‐glucose in the solution during extended deposition times of up to 300 s. Evaluation of the influence of porosity and electrospun PAN and PAN/PPy NFs on the formation of finely tuned dendritic Ag structures: g) FTIR of PAN and PAN/PPy mats with corresponding SEM images of dendritic Ag structures formed on the h) porous PAN/PPy NFs surface in contrast to the i) dense polymer network. j) SEM image displaying the porosity of PAN NFs and SEM images showing fine Ag dendrite formation on porous: k) PAN NFs and l) PAN/PPy NFs on Al foil.

Open circuit potential (OCP) measurements can serve as a descriptor of changes in potential metal pair interactions and electrolyte concentrations. OCP monitoring was carried out to evaluate the influence of D‐glucose and the GD reaction, complemented by SEM images of samples immersed in the freshly prepared Tollens’ reagent for the reaction time of *t* = 180 s (**Figure** **4**a–f). Samples with PAN NFs on Al foil in the presence of D‐glucose show controlled Ag dendrite growth by a moderate OCP rise at longer reaction times (Figure 4a,b). In contrast, in the absence of D‐glucose, a drastic OCP rise is associated with massive Ag outgrowth, observable in the corresponding SEM images, showing large Ag dendrites and particles (inset in Figure 4c).

Figure 4d–f shows the resulting OCP monitoring of a blank Al foil with and without the aid of D‐glucose (Figure 4e,f). Without D‐glucose, the reaction on PAN NF/Al is driven by the galvanic exchange between the redox pair, causing a potential drop due to electron supply from the Al foil in an alkaline solution. OCP declines over time in both cases to about −0.8 V versus Hg/HgO reference electrode. Initially, the rapid potential drop is due to electron release from Al oxidation, followed by the GD reaction where Ag^+^ ions reduce and deposit on the Al surface. This influences the metal ion concentration, affecting the OCP similar to the Al foil without PAN fibers (Figure 4d). The presence of PAN fibers increases the OCP, correlating with Ag dendrite growth (Figure 4a,c). Larger dendrites and particle agglomerates form without glucose, indicating the main role of D‐glucose and in addition PAN nanofibers in controlling Ag growth. This is supported by the steady‐state OCP observed for PAN NFs/Al, in contrast to a drastic potential rise in other cases (Figure 4a,c).

To prove the premise that a porous electrospun fiber network can serve as a template to mediate the controlled growth of Ag dendrites, we exchanged PAN with PAN/polypyrole (PPy) mats (Figure 4g). The chemical composition of PAN/PPy is very similar to aged PAN, as confirmed by FTIR analysis. To study the applicability of the proposed surface functionalization method, we selected PPy due to its chemical stability in alkaline solutions. Electrospinning of pure PPy is not possible due to its low solubility and its electroconductivity that potentially disrupts electrospinning. We used a mixture with PAN, 1.2 wt% PPy added into 8 wt% PAN dissolved in DMF to create electrospun PAN/PPy NF composites. Our study focused on the effect of porosity by comparing very porous and very dense fiber mat areas on Al foil (Figure 4h,i, respectively). To note, SEM images represent the PAN NFs/Al or PAN PPy NFs/Al immersed into Tollens’ reagent for the same reaction time, *t* = 180 s. We demonstrate the growth of Ag dendrites exclusively in the presence of porous PAN/PPy surfaces, in contrast to dense fiber mats showing no Ag structure (Figure 4i).

The hydrophobicity of PAN may inhibit the solution's access to the Al foil; however, wetting of the surface beneath the electrospun fibers during the experimental period cannot be excluded. The Al foil plays a crucial role in initiating the nucleation of Ag from Ag^+^ in the solution. The formation of silver particles or dendrites strongly depends on the surface treatment of the Al foil and the addition of D‐glucose (Figure S2, Supporting Information). While the polymer fiber network acts as a physical barrier, limiting access to the Al foil, the addition of D‐glucose primarily contributes to the fine growth of Ag dendritic structures governed by the interplay between GD and D‐glucose oxidation during silver reduction. We assume that fibers sterically slow down the diffusion of Ag ions by accommodating their access to the foil.

The back side of the immersed Al foil reveals Ag structures mainly in the form of irregular agglomerates, with only some dendrites present (Figure S3, Supporting Information), suggesting that the dendrites can be also formed on Al foil without a polymer template. The porous fiber network contributes to the growth of dendritic structures by guiding the growth of Ag building units between the fibers; however, in the case of thicker fiber mats electrospun for longer times, the dense polymer network obstructs the development of Ag structures, further emphasizing the critical role of the Al foil in synthesizing dendritic Ag structures.

The porosity of electrospun PAN NFs was determined using the ImageJ software. SEM images were converted to grayscale, and at specific gray shades, the cutoff was selected to distinguish fibers from pores. The percentage of the area within this threshold was calculated to determine the porosity.^[^ ^32^ ^]^ This microscopy method was preferred over other methods like Brunauer–Emmett–Teller gas adsorption (best suited for nanometer‐scale pores)^[^ ^33^ ^]^ for its ability to directly visualize porosity with the large pore distribution of interest for Ag dendrite fabrication. The porosity analysis reveals that dendrites only develop through the fiber polymer network when the porosity reaches at least ≈35% (Figure 4j), which is the case for the samples electrospun for 10 min on Al foil and taken from the edge of the PAN mat. In contrast, PAN NFs electrospun for the same time but taken from the middle with the highest fiber density do not accommodate the growth of Ag dendrites.

We used a solution of AgNO_3_ and NaOH in equal volumes. Unlike typical Tollens reagents (e.g., 0.1 M), we utilized a lower AgNO_3_ concentration (<0.05 M) for the synthesis of Ag dendritic structures. The solution was stirred to ensure rapid transport of reactants. By varying the immersion time from 60 to 180 s, similar dendritic Ag structures on the Al foil were formed (Figure S4–S6, Supporting Information). Moreover, when the porous PAN nanofibers were supported on nonmetallic carbon instead, only randomly distributed Ag aggregates resulted (Figure S7, Supporting Information).

The concentration of Ag^+^ ions in the AgNO_3_ solution was also varied to show the formation of larger silver dendrites. In this case, a higher AgNO_3_ initial concentration, in equal total volume of Tollens’ regent (i.e., 0.1 M AgNO_3_), leads to Ag structures consisting of a larger main stem and second‐order dendritic structures resulted (Figure S8, Supporting Information).

This study represents, to the best of our knowledge, the first report on the utilization of electrospun PAN nanofibers to accommodate the growth of Al‐anchored, highly stable Ag dendrites.

### 2.3 The Case of Ag Single Crystalline Dendrite Formation Based on the Interplay between GD and Autocatalytic Reaction

Our findings suggest a concurrent autocatalytic, glucose oxidation reaction in Tollens’ reagent to support the formation of single crystalline dendrites by accommodating the concentration and growth of Ag‐building units. The adsorption of D‐gluconic acid onto Ag structures can influence the reactivity and functionality in different ways: D‐gluconic acid can act as a stabilizing agent, adsorbing onto the surface of Ag structures to form a passivating layer; it can influence the nucleation and growth processes by adsorption on growing Ag surfaces and can also form coordination complexes with Ag ions. Finally, the presence of gluconic acid can influence the optical properties of silver structures, such as plasmon resonance.

We propose that, in addition to the Al foil triggering the GD reaction, a concurrent autocatalytic reaction occurs simultaneously in the D‐glucose solution (Figure 3a and 4b). The combined effect of GD and the autocatalytic reaction leads to a unique mechanism and drives the system into diffusion‐controlled conditions far from equilibrium, enabling improved control over the growth of dendritic Ag structures.

**Figure 5:**
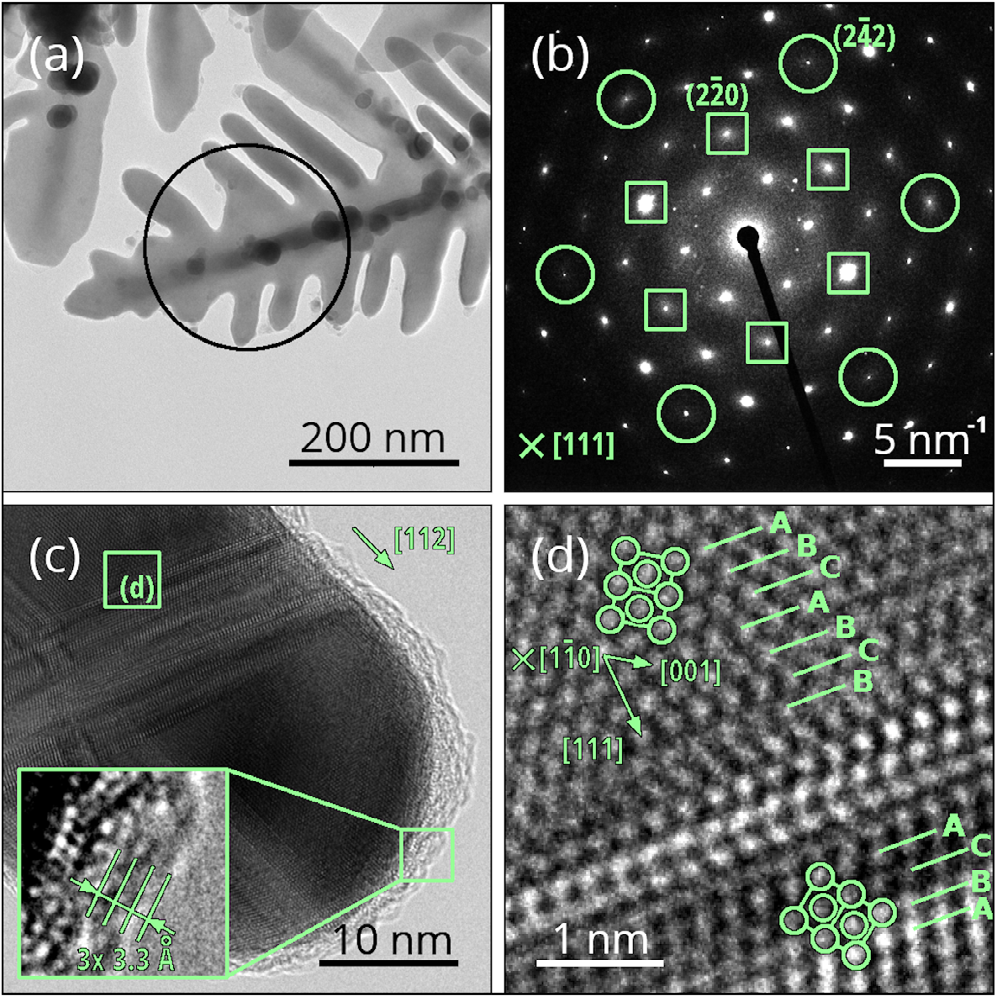
TEM analysis of the structure of Ag dendrites: a) 3D hierarchical Ag nanodendritic structures. b) The corresponding TEM diffraction pattern taken from a selected area indicated in (a) showing that the Ag dendrites have a crystalline FCC structure. The diffraction pattern is indexed for an FCC structure along [111] zone axis;{224} reflections corresponding to <112> growth directions of branches in (a) are marked in the pattern by circles, reflections of {220} type are indicated by squares. The additional 1/3{224} reflections are a consequence of the high density of faults present in the FCC structure. c) HRTEM image of the growing edge of a single branch, showing defects in the Ag crystal and the presence of a surrounding carbon layer (carbon interlayer spacings in the inset); the [112] growth direction is indicated. d) Image detail taken from the labeled region marked in (c); ABC stacking order with a stacking fault, crystal orientation, and FCC unit cells of the crystal matrix and a (111) twin are indicated.

**Figure 6:**
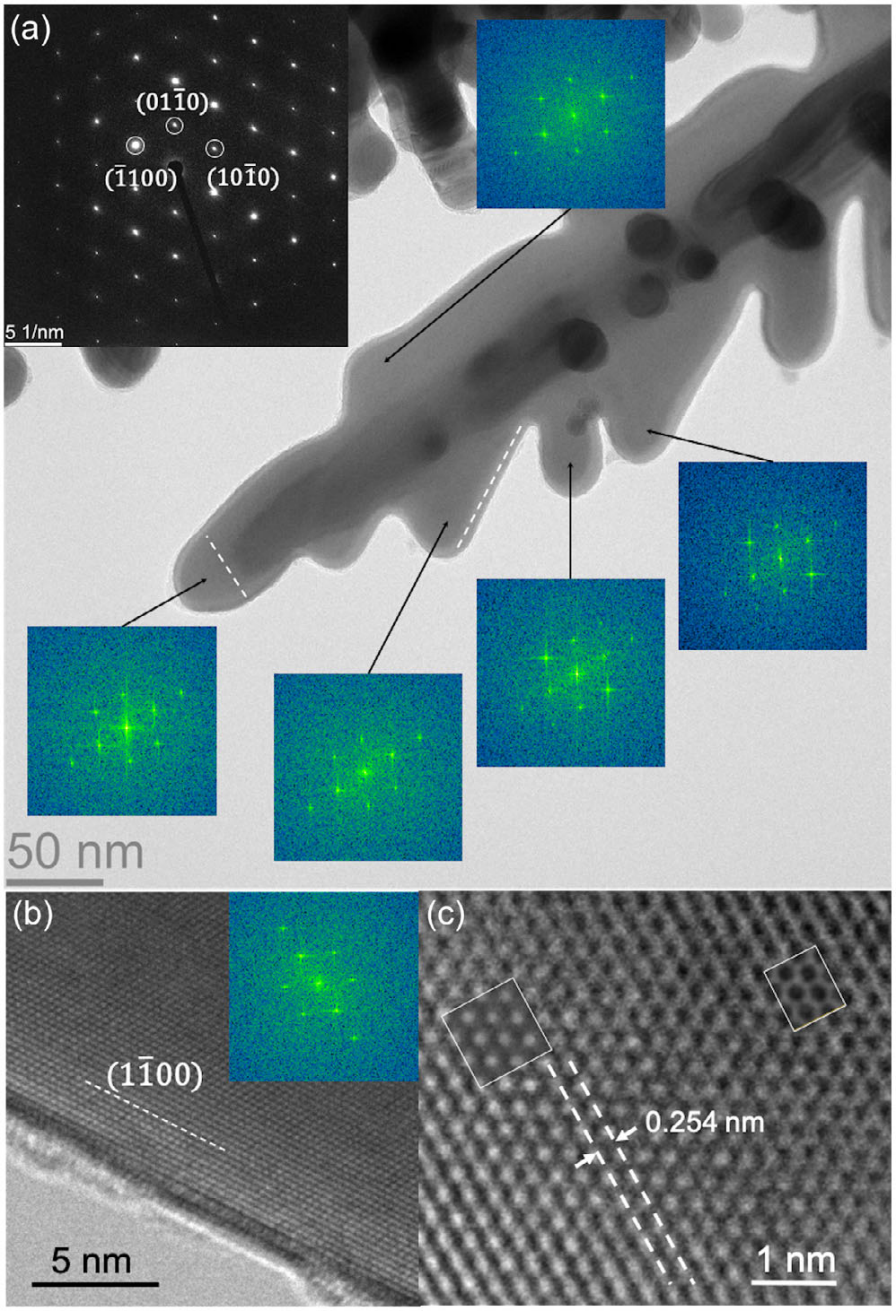
TEM analysis of an individual single crystalline Ag nanodendrite with HCP structure. a) TEM bright‐field image and the corresponding selected area diffraction pattern of the Ag nanodendrite. The diffraction pattern recorded from a large part of the nanodendrite shows a hexagonal pattern with strong reflections at positions related to {$1\bar{1}00$} planes of the hexagonal atomic structure (planar spacings 0.254 nm). FFT images calculated from HRTEM images of the different branches display the same hexagonal pattern. Their identical orientations disclose the single crystallinity of the whole Ag nanodendrite. The {$1\bar{1}00$} planes (indicated by dashed lines) are related to the growth direction and the edges of branches. b) HRTEM image showing the ($1\bar{1}00$) plane parallel to the edge of the nanodendrite. c) HRTEM image of an Ag nanodendrite close to the edge. The contrast variation in the HRTEM image with increasing thickness (from left to right) agrees well with image simulations of HCP Ag (shown as inset); beam direction is parallel to [0001].

**Figure** **5** and **6** depict the results of TEM investigations of fine Ag dendrites produced through PAN NF template‐mediated growth on an Al foil over 180 s in Tollens’ reagent. In this case, the lower concentration of AgNO_3_ led to very finely branched Ag dendrites with increasing surface coverage on the Al foil for prolonged reaction time.

In Figure 5a and in the supporting material (Figure S9, Supporting Information), the bright‐field images of Ag dendrites removed from the support show that the dendritic structure is well developed and its hierarchical morphology exhibits very fine branches (≈30 nm in width). SEM image of a tilted sample provides a more detailed view of the fine Ag dendritic structures formed between PAN fibers on the Al foil (Figure S10, Supporting Information). Detailed microscopic analysis from the diffraction pattern (Figure 5b) and the stacking sequence in atomic resolution images (Figure 5c,d) reveals a highly faulted crystalline FCC structure. Based on the analysis of the orientation of the diffraction pattern, the branches of the dendrite grow along <112> directions in the present case.

This is also confirmed by HRTEM images, showing the [112] direction along the edge of the branch. The appearance of additional 1/3 {224} reflections in the pattern can be attributed to a high density of thin HCP lamellae in the FCC crystal structure caused by stacking faults or twinning (Figure 5d), in agreement with recent reports.^[^ ^34^, ^35^ ^]^

The analysis of the thin surface layer reveals the presence of Ag_2_O and carbon, where the carbon structure is comprised of multiple layers. The existence of a thin layer of Ag_2_O can be ascribed to the partial oxidation of Ag dendrites during the preparation process, possibly during a brief rinsing of the specimens in distilled water. The HRTEM image indicates carbon layers surrounding the tips of nanobranched silver.

Although rare, the HCP structure in anisotropically grown Ag nanostructures has been found and achieved through wet chemical synthesis and electrodeposition methods.^[^ ^36^, ^37^, ^38^ ^]^ Figure 6 shows the analysis of another dendrite confirming its single crystallinity with HCP structure by combining diffraction pattern, HRTEM images, and corresponding FFT (Fast‐Fourier Transform) images. Both, FFT images calculated from the experimental HRTEM images of different branches and the electron diffraction pattern selected from a large part of the dendrite show the same orientation of a hexagonal pattern (Figure 6a). The high intensity of the inner spots of the diffraction pattern (indicated by {$\bar{1}100$} in the inset in Figure 6a) combined with experimental HRTEM images and their simulations (Figure 6b,c) reveals the HCP atomic structure. The HRTEM image simulations based on the HCP of a branch with increasing thickness (from left to right) are shown as rectangular inset in Figure 6c and agree well with the experimental HRTEM image. The growth direction of the main branch as well as some edges of the side branches are related to {$1\bar{1}00$} planes as indicated by the white dashed lines in Figure 6a,b. Zhou et al. suggested that the formation of the high‐energy HCP phase in silver may be facilitated by the carboxylic group (−COOH) derived from the formic acid, which is produced during the oxidation of aldehyde groups present in the solution^[^ ^39^ ^]^ Our findings align with this observation, as the carboxylic group is the characteristic functional group in D‐gluconic acid, which is generated during the oxidation of D‐glucose.

We compare this Ag dendrite fabrication method to other techniques, such as electrodeposition, which relies on the concentration of the electric field at convex surfaces induced by the applied potential and diffusion‐controlled conditions.^[^ ^40^ ^]^ The process of GD can be also seen as an electrochemical system consisting of multiple cathodes (reduction of the less noble metal salt into the metal atoms) and anodes (oxidation of the less noble metal leading to dissolution).^[^ ^24^ ^]^ Electrons generated at the anode are then consumed at the cathode where Ag dendrites develop. Thus, it resembles a self‐sustained system driven by the redox pair potential difference, like electrosynthesis but without the use of external current. Similarly, we assume that galvanic coupling can be seen as a process where the electric field concentrates around the positions where aluminum is oxidized and silver cations are reduced to metallic silver (Figure 3). The Ag grows faster at steps or protrusions leading to fast depletion of available Ag^+^ ions.

To reveal the role of D‐glucose in the mechanism of the formation of Ag dendritic structures with lower AgNO_3_ concentration, that is, 0.03 M, we compared the results of Ag dendritic structures formed on Al/PAN NFs without glucose in the solution to those formed with increasing amounts of D‐glucose, using a synthesis time of 60 s in all cases (Figure S11, Supporting Information). SEM images reveal that the locations of Ag dendrite formation (determined by the Al foil) are consistent with results based on GD. However, there is a noticeable reduction in dendrite size with a higher amount of D‐glucose. This indicates that D‐glucose primarily influences the growth rate of Ag dendrites, while the porous PAN network sterically hinders the access of Ag^+^ to the Al foil. The Ag precursor can adsorb onto the growing surface by the autocatalytic process with the aid of D‐gluconic acid that serves as a capping agent on the growing Ag dendrites. The concurrent autocatalytic reaction ensures diffusion control conditions consuming available Ag^+^ ions, while the electrospun PAN network restricts the availability of Ag^+^ ions and serves as a physical barrier on the Al foil with a few nucleation sites (Figure S11, Supporting Information).

In the case of higher concentration gradients, that is, at higher AgNO_3_ concentrations, larger dendrites form (Figure S8, Supporting Information). Although the exact mechanism of dendrite growth is still uncertain, our results confirm the role of D‐glucose in the development of Ag dendritic structures. This synergy between GD and autocatalytic reaction on the formation of Ag dendrites is illustrated by the absence of galvanic coupling (Figure S1a,b, Supporting Information) and the absence of D‐glucose as a reducing agent (Figure S1c,d, Supporting Information).

**Figure 7:**
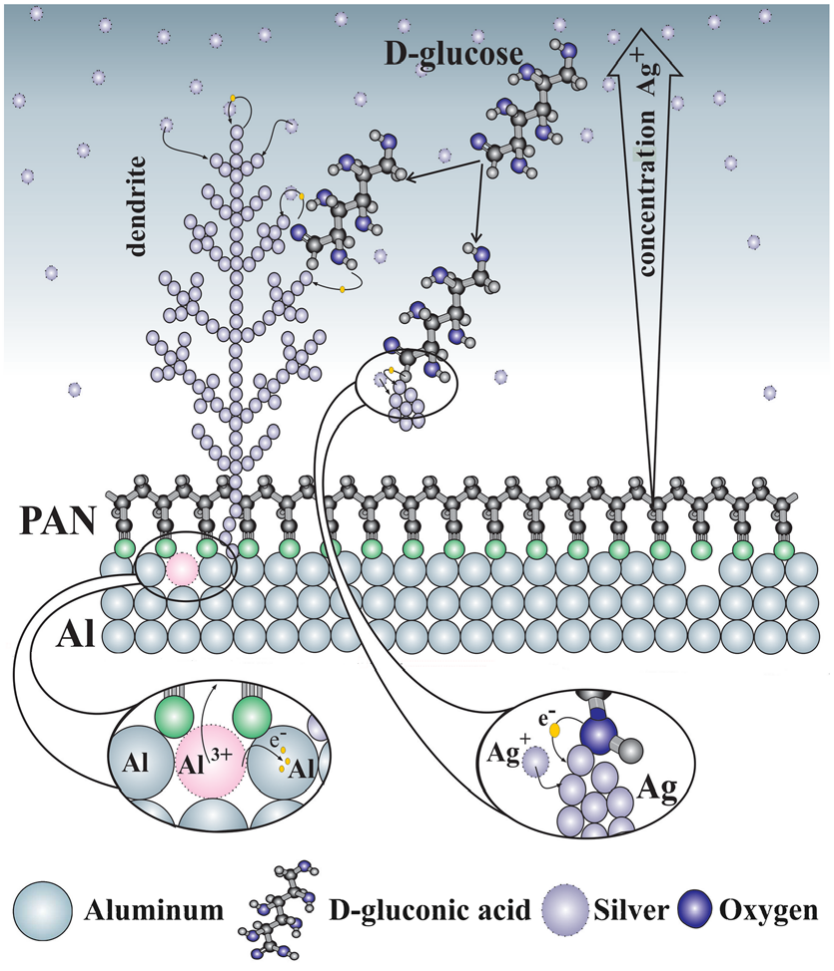
Proposed mechanism of PAN NFs/Al template‐mediated growth of finely branched dendritic Ag structures based on the interplay between galvanic displacement and glucose oxidation reaction, suggesting simultaneous Ag stabilization by D‐gluconic acid adsorption.

The proposed mechanism of Ag dendritic growth is depicted in **Figure** **7**. The Al foil serves as a classical anode material that undergoes oxidation in the solution under Reaction (4) and (5), forming a flux of released e^−^ interacting with Ag^+^ ions forming Ag, nucleating preferentially at specific locations on the Al foil and acting as cathode (Figure 3, S12, and S13, Supporting Information): moderate supersaturation drives nucleation and growth thermodynamically away from equilibrium, yielding diffusion conditions^[^ ^41^, ^42^ ^]^ facilitated by limited accessibility of the support surface intertwined by the PAN network and a concurrent autocatalytic process, reducing the available Ag^+^ concentration for direct GD reaction.

Similar to the work of Zhou et al. in controlling the growth of silver‐like flower structures using aldehydes,^[^ ^36^ ^]^ we suggest a striking role of glucose in the growth of Ag dendritic surfaces by adsorption of Ag building units and D‐gluconic acid formed as a result of D‐glucose oxidation during the reduction of Ag^+^ ions in the solution.

In brief, this synthesis method enables high‐yield, room‐temperature production of stable Ag single crystalline dendrites in a very short time. Adding D‐glucose to the solution enhances control over dendritic growth on both untreated and templated Al foil. Overall, this approach simplifies Ag dendrite synthesis, surpassing complex multistep methods while offering improved growth rate control through D‐glucose addition. Furthermore, this work stands out as an experimental example of GD, serving as a bridge between the chemical and electrochemical approaches proposed by Xia and coworkers.^[^ ^24^ ^]^

#### 2.3.1 Stability of the Fabricated Ag Dendrites

The main drawback in the use of Ag structures as an effective SERS substrate is exposure to the ambient atmosphere, leading to the evolution of an oxidation layer. Particularly, in the case of Ag dendrites that proved to be very advantageous SERS substrates, the high reactivity of the branched surface leads to pronounced contamination and oxidation. This process results in a change in dendrite shape and a decrease in effectiveness over time. Many strategies were introduced to stabilize the Ag dendrites; formation of bimetallic structures is one valuable approach.^[^ ^43^, ^44^ ^]^ Another study introduces Ag nanoparticle protection by the formation of the ultrathin carbon layer.^[^ ^45^ ^]^ Recent work employs atomic layer deposition of ZnO on Ag dendrites to achieve to improve their SERS sensitivity and thermal stability.^[^ ^17^ ^]^

In our study, we assume the adsorption of D‐gluconic acid that is formed as a by‐product of glucose oxidation upon Ag^+^ reduction, acting also as a stabilizing agent. Although known in the capping of Ag nanoparticles,^[^ ^46^ ^]^ this study is the first report of its use in the fabrication of dendritic Ag structures. To prove this assumption, we carried out a series of SERS experiments on different sample locations, on freshly prepared samples in the presence and absence of glucose. A maximum laser power of ≈0.7 mW and accumulated energy of ≈70 mJ per sample position were sufficient to detect D‐gluconic acid on Ag dendrites, whereas in ref. [47] a laser power of 440 mW and an accumulated energy of 23 kJ was needed to acquire a Raman spectrum of D‐gluconate in solution.

**Figure 8:**
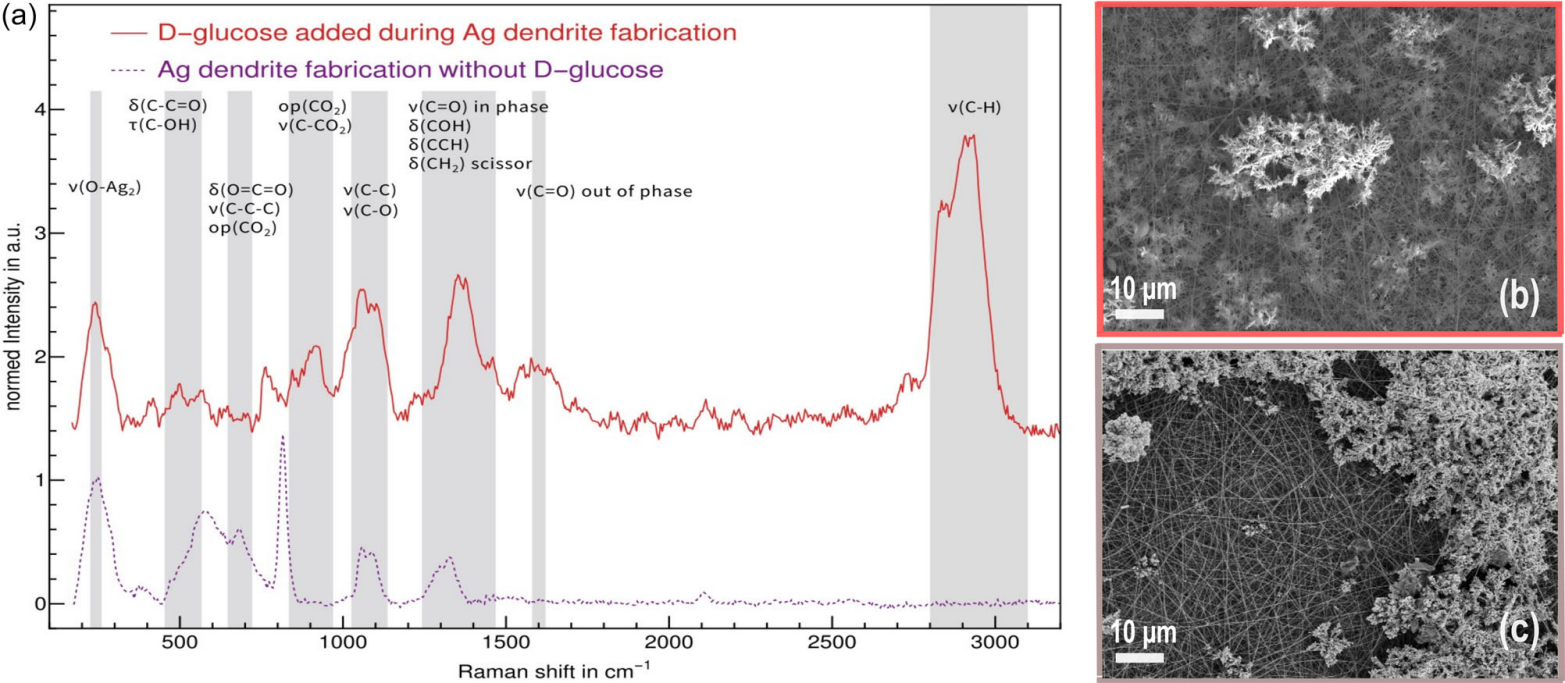
a) Raman spectra at Ag dendrite positions for different samples; The spectra are normalized by the Ag peak corresponding to the O‐Ag_2_ stretching vibration. Highlighted vibrational modes (*υ*: stretching, *δ*: bending, *τ*: torsion, op: out of plane) of D‐gluconate and D‐gluconic acid compared to literature.^[^ ^46^, ^47^ ^]^ SEM images of Ag dendrites formed in the: b) presence and c) absence of D‐glucose in the solution.

We compared the location of the Raman peaks of D‐gluconate and D‐gluconic acid found in literature^[^ ^46^, ^47^ ^]^ with those measured on our samples. D‐gluconate and D‐gluconic acid differ by one hydrogen atom and should be indistinguishable for our Raman results. As shown in **Figure** **8**, characteristic vibrational modes like the C—H stretching vibrations in the region 2800–3100 cm^−1^ and the C=O out of phase (asymmetric) stretching vibrations at ≈1600 cm^−1^ are only present in samples fabricated with the addition of D‐glucose. For these samples, the stretching and bending vibrations of C, O, and H compounds above 1000 cm^−1^ are enhanced relative to samples prepared in the absence of D‐glucose in the solution, thus relying on the galvanic displacement mechanism of Ag formation only.

Among the various synthesis methods of Ag dendritic structures known to achieve excellent SERS sensitivity of chemical and biological analytes down even to the level of a single molecule,^[^ ^15^, ^48^, ^49^, ^50^, ^51^, ^52^ ^]^ we focus on the role of D‐glucose in interplay with galvanic displacement. The role of D‐gluconic acid can be manyfold: it can contribute to the synthesis of single crystalline Ag dendrites by controlling their size, acting as a capping agent, or participating in a complexation reaction with Ag, thereby influencing the growth process. Our primary aim is to prove the SERS of D‐gluconic acid as an indication of its role as a capping agent for silver dendrites. SERS measurements detect the presence of D‐gluconic acid on Ag dendrites (Figure 8), aligning with HRTEM findings (Figure 5 and 6) showing the presence of carbon layers on Ag branches. It should be noted here that Raman detection of glucose is a challenging task due to small scattering cross sections.^[^ ^53^ ^]^

To further prove the presence of the D‐gluconic acid, we support Raman findings by comparing the results acquired by the FTIR method. Despite the high background signal resulting from the use of Al foil, FTIR analysis shows a small intensity of characteristic functional groups that can be attributed to D‐gluconic acid (Figure S14, Supporting Information).

Despite being one of the most promising SERS substrates, the degradation of silver that undergoes atmospheric corrosion is an important factor that impedes its long‐term use.^[^ ^17^, ^44^, ^54^ ^]^ It was shown that when SERS substrates are exposed to ambient air, its degradation results in rapid decay of SERS signal, limiting its further applicability.^[^ ^54^ ^]^ In this context, the SERS signal of D‐gluconic acid in our study serves as an indicator of the stability of Ag dendrites. Additionally, it demonstrates higher sensitivity compared to that obtained from Ag nanoparticles capped with D‐gluconic acid.^[^ ^46^ ^]^ Its detection in Raman spectra even after 1 year suggests the enhanced stability of the Ag structures over time. SEM image reveals the preserved structure of Ag dendritic branches, showing only minor surface segregation and low levels of oxidation even after up to 1 year (Figure S15 and S16, Supporting Information).

It should be highlighted that the samples were kept under ambient condition during this period. Raman spectra acquired on the sample 1 year after sample fabrication show the presence of D‐gluconic acid (Figure S16, Supporting Information). We assume two dominant factors for the observed stability: 1) the formation of a single crystalline structure without grain boundaries that, in contrast to polycrystalline structures, minimizes diffusivity and possible corrosion and 2) a partially protective role of the D‐gluconic acid layer covering the dendrites and suppressing conceivably reactivity of Ag with the atmosphere. A significant advantage of the proposed approach is foreseen for use as flexible membrane substrates, usually requiring multiple processing steps, showing poor anchoring, or suffering from loose hierarchy. The combination of chemical and electrochemical reactions to produce Ag dendritic structures presents an avenue for further exploration, particularly through the choice of substrate or surface treatment.

## 3 Conclusion

We show the controlled growth of finely branched dendritic Ag structures on an Al foil, mediated by an electrospun PAN template and the addition of D‐glucose. The interplay between concurrent galvanic displacement and autocatalytic electroless reactions is shown to be crucial for the outgrowth of Ag dendrites. We achieve well‐developed, hierarchical branches of synthesized Ag dendrites with very fine branches (≈30 nm) exhibiting a highly faulted single crystalline FCC structure. A significant advantage of this approach is its potential use as flexible membrane SERS substrates, which typically require multiple processing steps, exhibit poor anchoring, or may exhibit a loose hierarchical structure, which can negatively impact their performance. A key benefit of this synthesis method is the fabrication of stable single crystalline Ag dendrites protected by an organic layer of D‐gluconic acid, which are able to retain their fine dendritic structure even after 1 year under ambient conditions.

## 4 Experimental Section

4.1

4.1.1

##### Synthesis of PAN Nanofiber Mats

The synthesis of PAN nanofibers by electrospinning was carried out by adjusting the electrospinning parameters and the concentration of PAN in *N*,*N*‐dimethylformamide (DMF). We selected conditions that yield a uniform, bead‐free PAN nanofiber matrix by selection of factors such as optimum PAN concentration, a tip‐to‐collector distance set to 12 cm, a solution feeding rate of 0.2 mL h^−1^, and an applied voltage of 7.5 kV, with the latter two playing the dominant role. The solution used for electrospinning was made by dissolution of 8.5 wt% PAN (Sigma–Aldrich, molecular weight 150, 000) in DMF, stirred for 2 h at 40 °C. Synthesis of PAN/ PPY NFs by electrospinning was carried out by adjusting electrospinning parameters with a tip‐to‐collector distance set to 15 cm, a solution feeding rate of 0.15 mL h^−1^, and an applied voltage of 10.0 kV. The solution used for electrospinning was made by grinding 8.0 wt% PAN (Sigma–Aldrich) and 1.2 wt% PPY for 1 h, followed by dissolving the mixed powder in DMF, and stirring for 12 h at 60 °C. An Al foil was used as a counter electrode during electrospinning.

##### Fabrication of Ag Dendrites by a PAN Template‐Mediated Approach

Electroless, autocatalytic deposition was carried out in Tollens’ reagent: Ag_2_O was firstly formed in NaOH, according to Reaction (1) in the presence of AgNO_3_. In the next step, the formed Ag_2_O was dissolved in excess ammonia creating a diammine complex, or Tollens’ reagent (2). Finally, the electroless Ag deposition by reduction of Ag^+^ ions to Ag was enabled in the presence of D‐glucose that acted as a reducing agent, thus driving oxidation to D‐gluconic acid and providing electrons for reduction of Ag^+^ to Ag, according to Reaction (3).

(1) $$2{\text{AgNO}}_{3}+2\text{NaOH}\to {\text{Ag}}_{2}O+2{\text{NaNO}}_{3}+H_{2}O$$

(2)

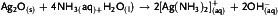



(3)

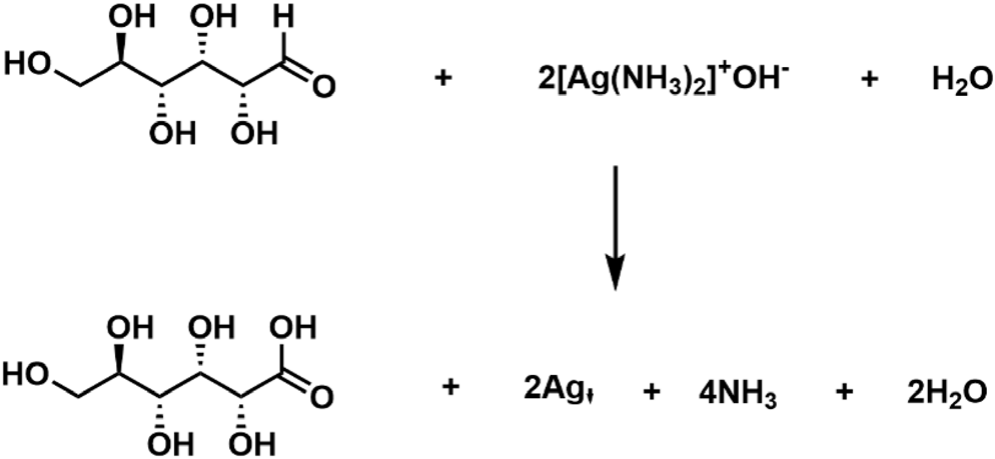




The GD reaction took place by the redox pair of Ag^+^ ions, present in the solution in the form of diamine complex, in the contact with Al substrate undergoing Reaction (4) and (5)

(4) $${3\text{Ag}}^{+}{+\;3e}^{-}\to 3\text{Ag}$$

(5) $$\text{Al }\to {\text{ Al}}^{3+}\;{+\;3e}^{-}$$

The balanced equation for the galvanic coupling Reaction (4) and (5) showed that 3 Ag atoms would be formed from one displaced Al atom undergoing the oxidation process.

In the presence of alkaline hydroxides such as NaOH, Al metal dissolved to form Na‐aluminate according to (6)

(6) $${2\text{Al }+\;2\text{ NaOH }+\;6H}_{2}\text{O }\to \;2\text{Na }\left[\text{Al}{\left(\text{OH}\right)}_{4}\right]{\;+\;3\text{ H}}_{2}$$

(7) $${\text{Al}}_{2}O_{3}+2\text{NaOH}\to {2\text{NaAlO}}_{2}\;{+\text{ H}}_{2}O$$

Besides, the amphoteric character of Al and its oxide Al_2_O_3_ that is commonly present as native oxide layer on Al allows dissolution in the presence of NaOH according to Reaction (7).

##### Characterization of Electrospun Fibers

FTIR measurements of the fiber mats collected on Al foil were carried out using ALPHA II FTIR spectrometer (Brucker Optics GmbH & Co. KG, Germany) in attenuated total reflectance (ATR) with a diamond crystal measurement interface. In a spectral range of 400–4000 cm^−1^, 24 scans with a resolution of 4 cm^−1^ were accumulated. A baseline correction was done using the software OPUS version 8.8.4 from Brucker Optics GmbH & Co. KG while band positions and absorption intensities were determined by Origin Pro.

Local Raman spectra at Ag dendrite positions in between the PAN polymer fiber mats were acquired at a WITec alpha 300 A using a red laser of 633 nm wavelength and a grating of 300 g mm^−1^. Multiple spectra from equivalent sample positions were averaged to avoid dynamic effects during SERS. Single acquisitions were performed at low laser powers of 0.1–0.7 mW and low accumulated energies (<70 mJ). The spectra were background subtracted before averaging using the Fityk software.^[^ ^55^ ^]^

The contact angle of electrospun fiber mats on Al foil was measured using a Kruss Drop Shape Analyzer, DSA 100 (Germany) placing a drop of DI water of several μm on the PAN surface. To analyze the surface, topology, morphology, and composition of bare and Ag surface functionalized electrospun PAN mats, electron microscopy was used. An SEM, ZEISS SIGMA HD VP device, with imaging resolution as small as 1 nm, was employed to analyze as‐synthesized and functionalized mats. The samples were measured without additional coating. For the detailed structural characterization of the Ag nanodendritic structures, dendrites were scratched off from the surface of the Al foil onto a holey carbon grid, allowing to investigate multiple dendrites by transmission electron microscopy (TEM). The TEM studies were carried out using a JEOL 2200FS microscope operated at 200 kV equipped with a CMOS camera (TVIPS XF416) and EDX (Oxford AZtecEnergy UltimMax TEM) for chemical analysis. Bright‐field, dark‐field, and high‐resolution (HRTEM) images as well as selected area diffraction patterns of multiple areas were taken to characterize different areas of the dendrites in detail. HRTEM image simulations using JEMS^[^ ^56^ ^]^ were carried out in order to validate the structure and compare the contrast with that of experimental images.

## Conflict of Interest

The authors declare no conflict of interest.

## Author Contributions

**Lidija D. Rafailović**: conceptualization (lead); formal analysis (equal); investigation (lead); methodology (equal); validation (lead); writing—original draft (lead); writing—review and editing (lead). **Stefan M. Noisternig**: formal analysis (equal); investigation (equal); methodology (supporting); writing—original draft (supporting). **Jana Bischoff**: investigation (supporting). **Christian Rentenberger**: formal analysis (supporting); writing—review and editing (supporting). **Daniel Bautista–Anguis**: investigation (supporting). **Huaping Sheng**: investigation (supporting). **Christoph Gammer**: formal analysis (supporting). **Jia Min Chin**: writing—review and editing (supporting). **Adam Elbataioui**: investigation (supporting). **Huanqing Zhang**: investigation (supporting). **Jürgen Eckert**: funding acquisition (lead); writing—review and editing (supporting). **Tomislav Lj. Trišović**: conceptualization (equal); writing—review and editing (equal).

## Supporting information

Supplementary Material

## Data Availability

The data that support the findings of this study are available from the corresponding author upon reasonable request.
