# Supplementary material for: Enhanced Control of Single Crystalline Ag Dendritic Growth on Al Foil via Galvanic Displacement and Simultaneous Oxidation of D‐Glucose
Source: Small Sci. 2025 Jan 28;5(4):2400478. doi: 10.1002/smsc.202400478 (PMC12245025; doi:10.1002/smsc.202400478)
Supplement: Supplementary file 1 — Supplementary Material [file SMSC-5-2400478-s001.pdf]

# Supporting Information

## Enhanced Control of Single crystalline Ag Dendritic Growth on Al Foil via Galvanic Displacement and Simultaneous Oxidation of D-Glucose

*Lidija D. Rafailović[a,b]*, Stefan M. Noisternig[b,c], Jana Bischoff[b], Christian Rentenberger[c], Daniel Bautista - Anguis[d], Huaping Sheng[b], Christoph Gammer[b], Jia Min Chin[e], Adam Elbataioui[a], Huanqing Zhang[a], Jürgen Eckert[a,b]*, Tomislav Lj. Trišović[f]*

[a] Department of Materials Science, Chair of Materials Physics, Montanuniversität Leoben, 8700 Leoben, Austria

[b] Erich Schmid Institute of Materials Science, Austrian Academy of Sciences, 8700 Leoben, Austria

[c] Faculty of Physics, Physics of Nanostructured Materials, University of Vienna, 1090 Vienna, Austria

[d] Polymer Competence Center Leoben GmbH, 8700 Leoben, Austria

[e] Institute of Inorganic Chemistry – Functional Materials, Faculty of Chemistry, University of Vienna, 1090 Vienna, Austria

[f] Institute of Technical Sciences of the Serbian Academy of Sciences and Arts, 11000 Belgrade, Serbia

E-mail: lidija.rafailovic@oeaw.ac.at; juergen.eckert@unileoben.ac.at

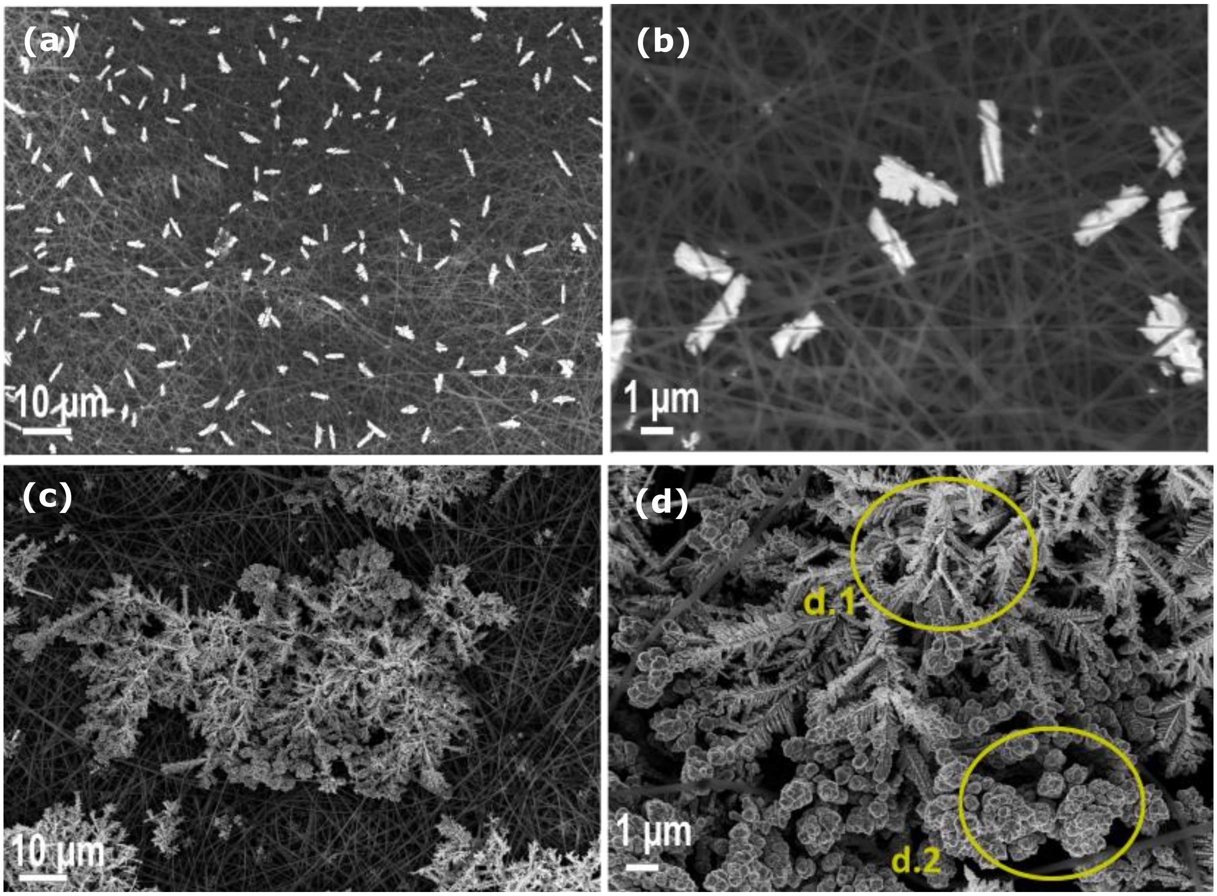


**Figure S1 (**a) and (b) SEM images highlighted in green showing Ag deposits in the form of irregular platelets on electrospun PAN nanofibers fabricated on filter paper and collected after 180 s; (c) and (d) SEM images highlighted in yellow showing mixed Ag deposition in the form of (d.1) large dendrites and (d.2) attached particles on electrospun PAN nanofibers on Al foil in the Tollens' reagent in the absence of glucose reagent clearly indicating that Ag dendritic growth is governed by galvanic coupling.

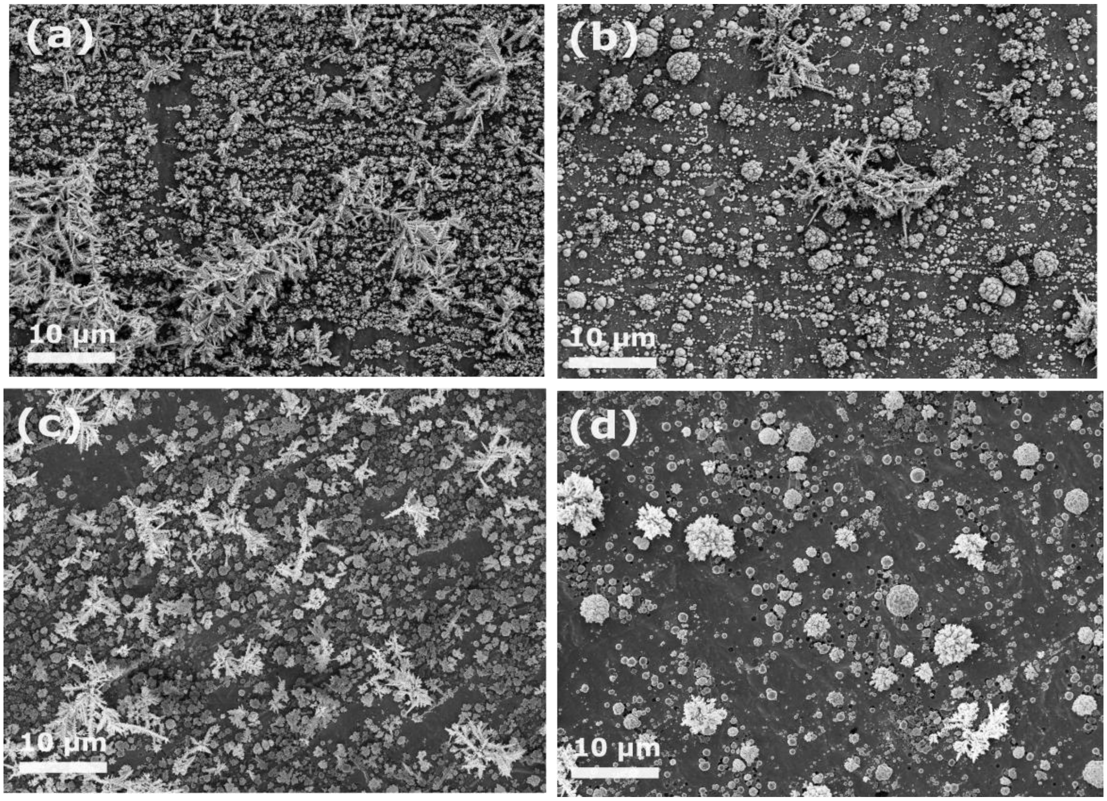


**Figure S2**. The differences in Ag growth on Al foils after immersion in the Tollens-based reagent for the same duration: Large, well-developed dendrites are observed ((a) and (b)) on surface treated and cleaned Al foils without addition of D-glucose. In contrast, the addition of D-glucose leads to only partial particle coverage and localized Ag growth evident at specific sites of alcohol cleaned Al foils ((c) and (d)).

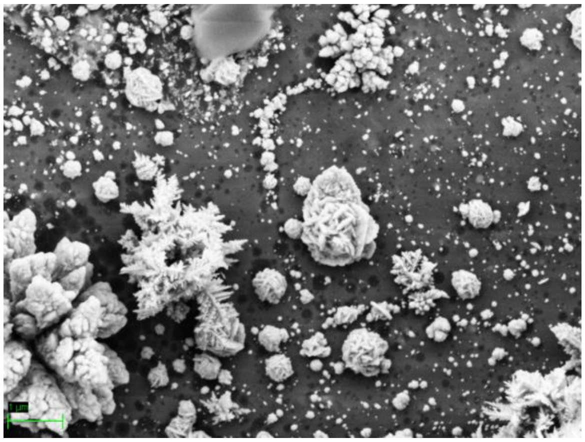

**Figure S3**. SEM image of irregular Ag structures grown on the back side of Al foil with PAN/PPy NFs (cf. Figure 4 (i), in the form of particles and dendrites.

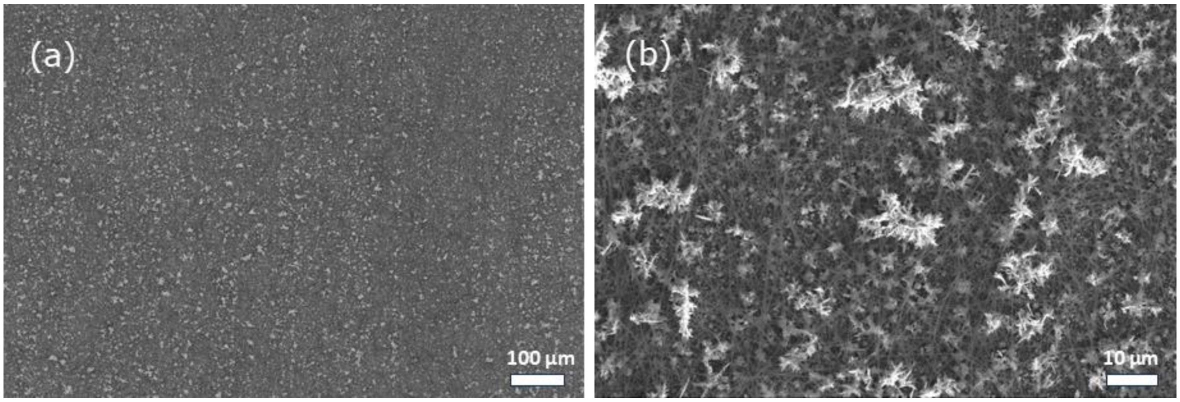


**Figure S4** SEM images of surface funtionalized and electrospun PAN template mediated Ag dendritic structures on Al foil immersed into Tollens' reagent for 60 s, taken at different magnifications.

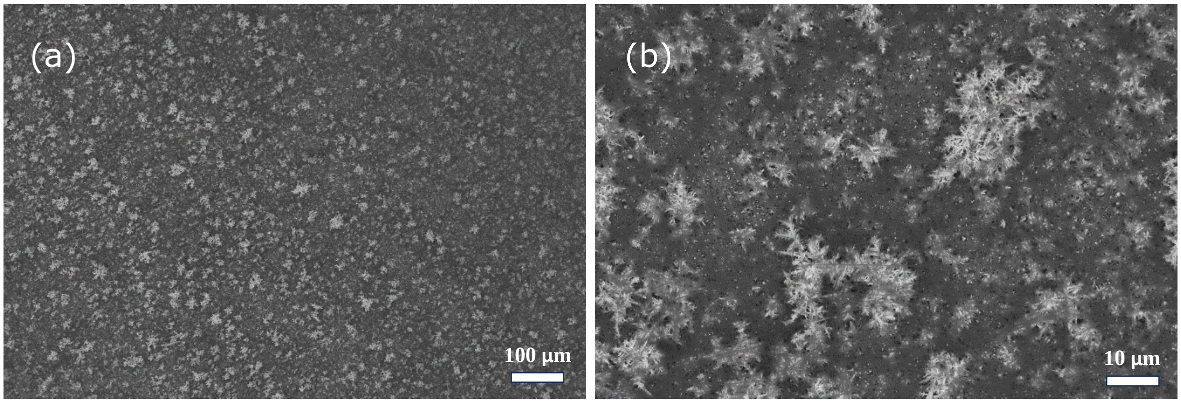


**Figure S5** SEM images of surface funtionalized and electrospun PAN template mediated Ag dendritic structures on Al foil immersed into Tollens' reagent for 120 s, taken at different magnifications.

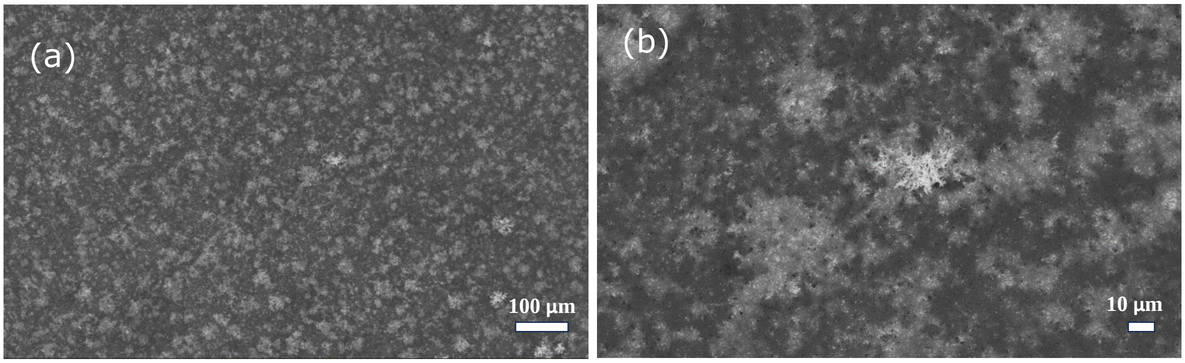


**Figure S6** SEM images of surface funtionalized and electrospun PAN template mediated Ag dendritic structures on Al foil immersed into Tollens' reagent for 180 s, taken at different magnifications.

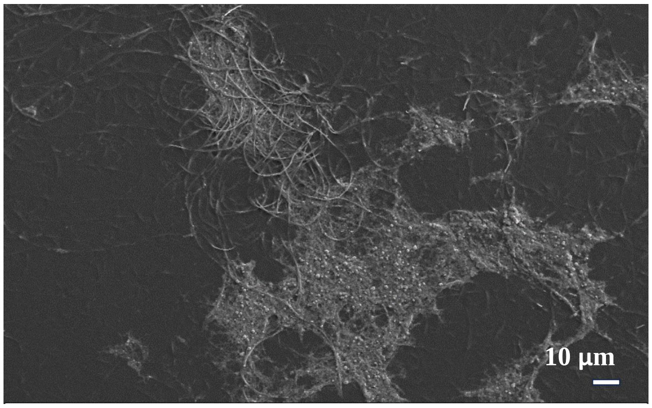


**Figure S7** SEM image showing only partlial coverage of electrospun PAN nanofibres by Ag on carbon support upon direct immersion into Tollens' reagent for 180 s.

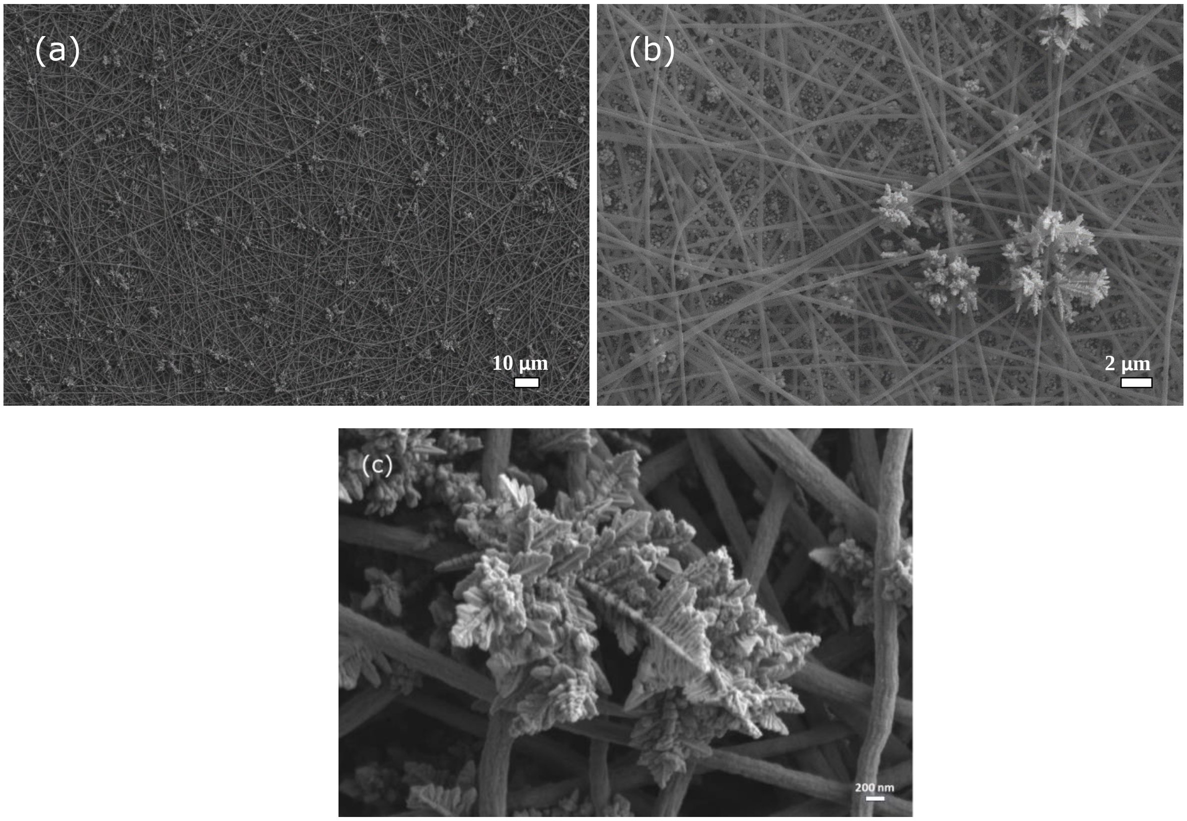


**Figure S8** SEM images of Ag dendritic structures obtained by template mediated electrospun PAN nanofibre network on Al foil. Fabrication was carried out by direct immersion into Tollens' reagent for 180 s in 0.1 M $AgNO_3$; SEM images are taken at different magnifications.

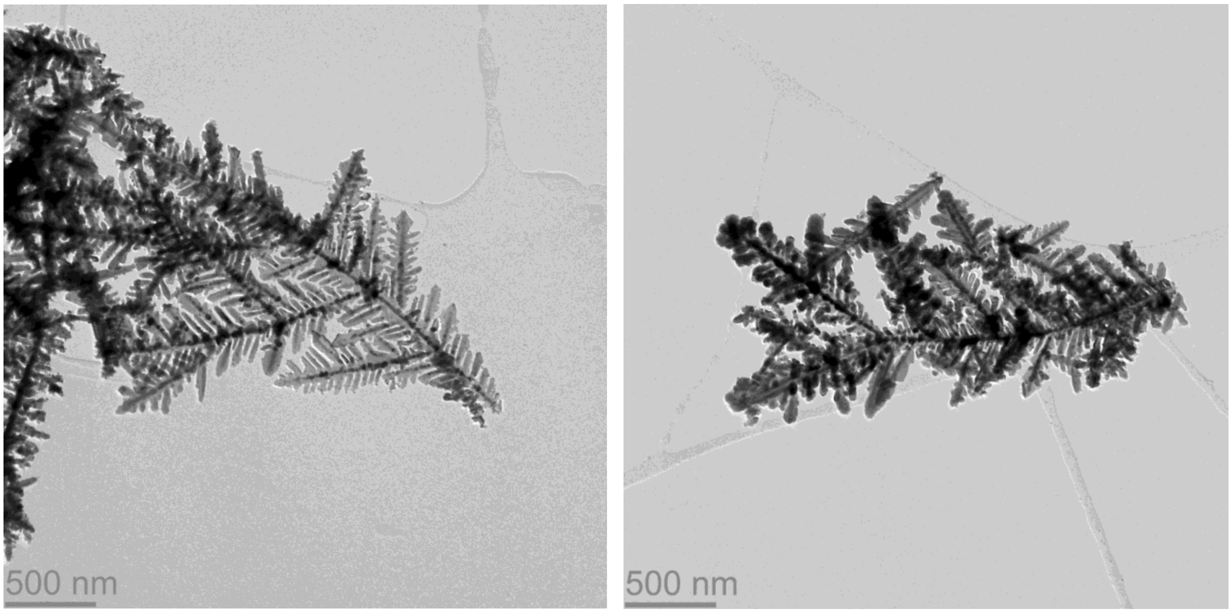


**Figure S9** The bright-field images of Ag dendrites removed from the support showing well developed hierarchical morphology of silver branches.

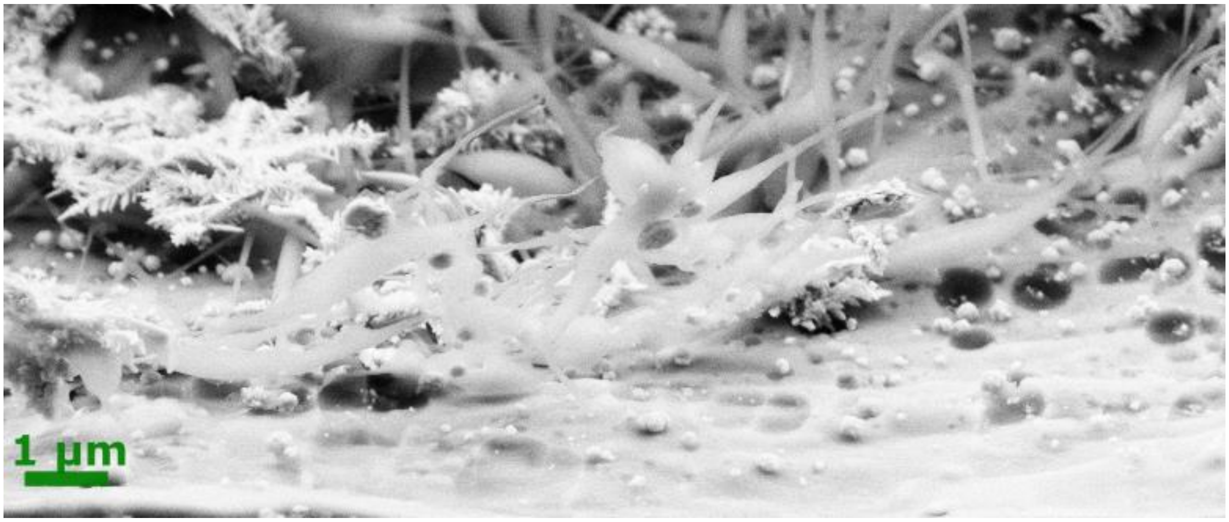


**Figure S10** SEM image of a tilted sample provides a more detailed view of the fine Ag dendritic structures formed between PAN fibers on the Al foil.

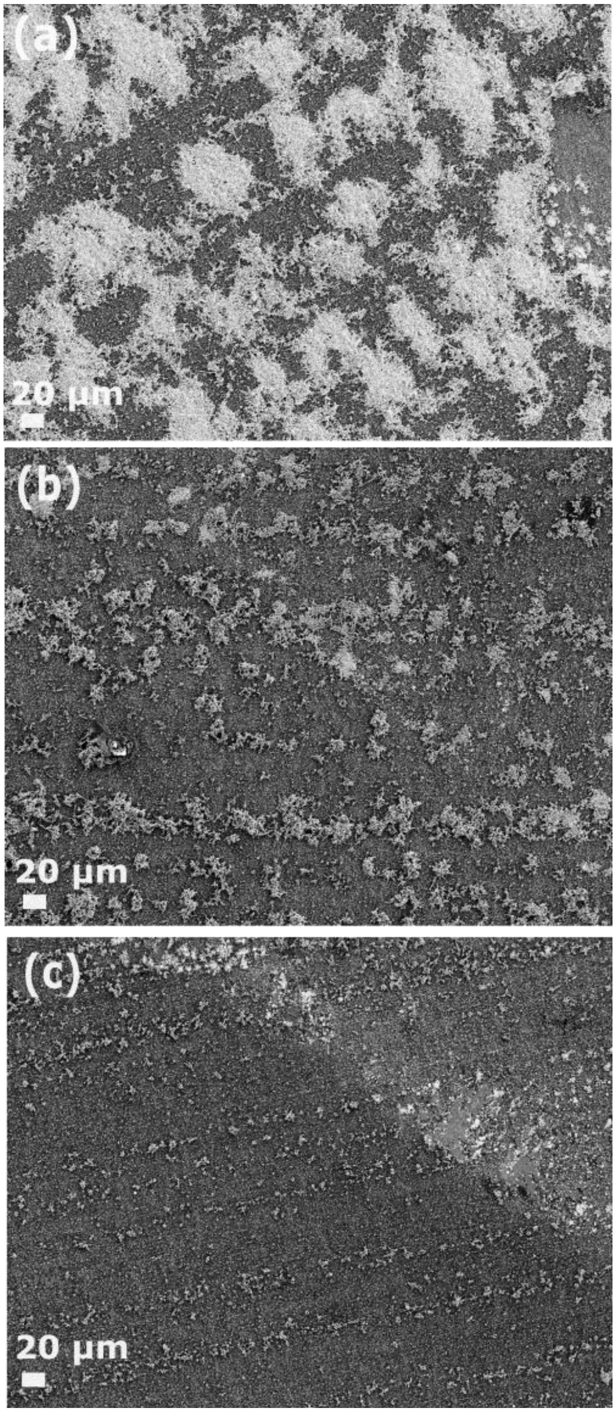


**Figure S11** SEM images showing the role of D-glucose in the mechanism of the formation of Ag dendritic structures on Al foil templated with PAN NFs: (a) without addition of D-glucose in the solution; and with increasing amounts of D-glucose: (b) 3ml and (c) 6ml of 0.25M D-glucose using a synthesis time of 60 s in all cases.

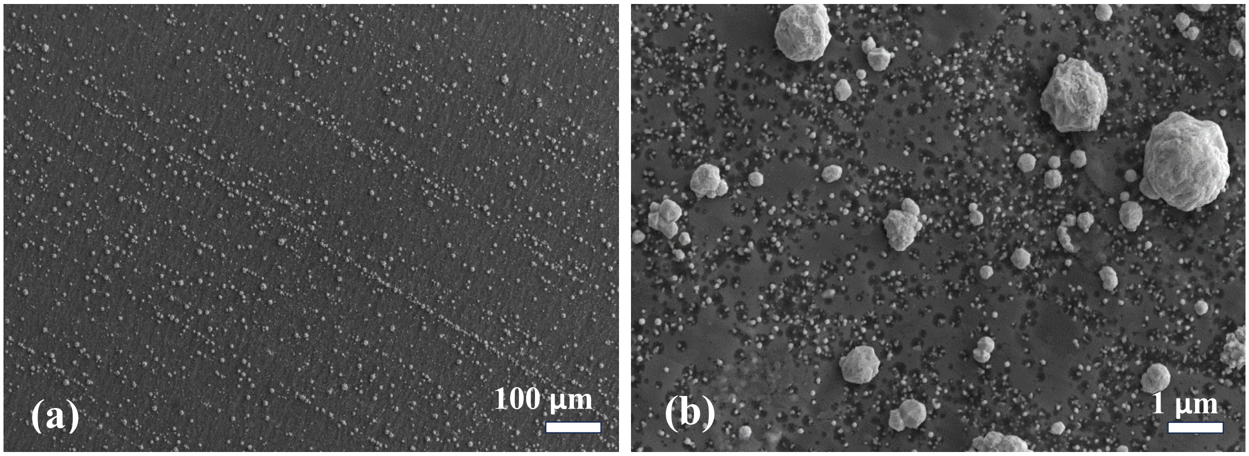

**Figure S12** SEM images of bare Al foil substrate immersed into Tollens’ reagent for 30 s showing preferential Ag deposition growth in form of Ag particles at specific locations with presumably thinner Al oxide layer.

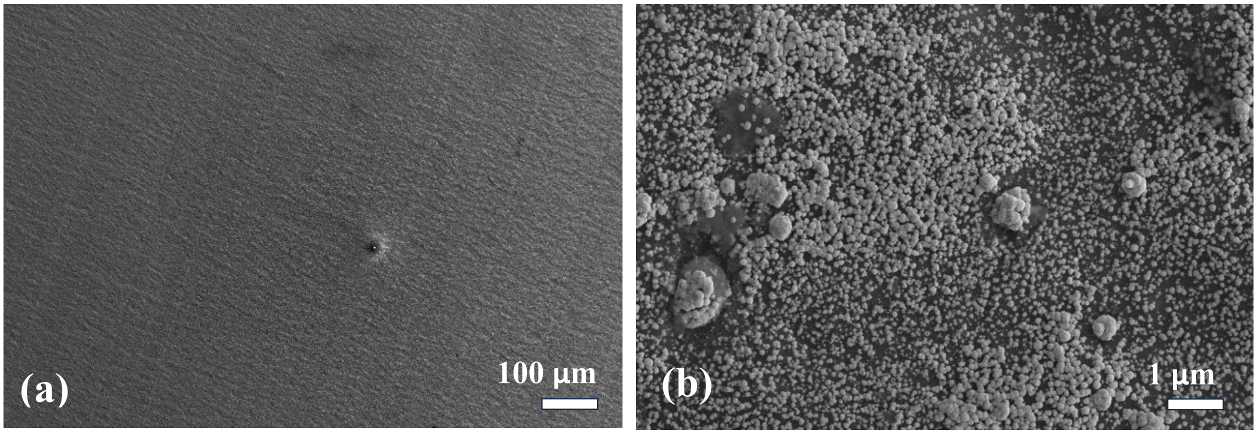

**Figure S13** SEM images of bare and etched Al foil substrate, washed and subsequently immersed into Tollens’ reagent for 30 s showing Ag particles formation with more unform coverage compared to untreated Al foil surface (cf. Figure S12) that is attributed to Al oxide removal.

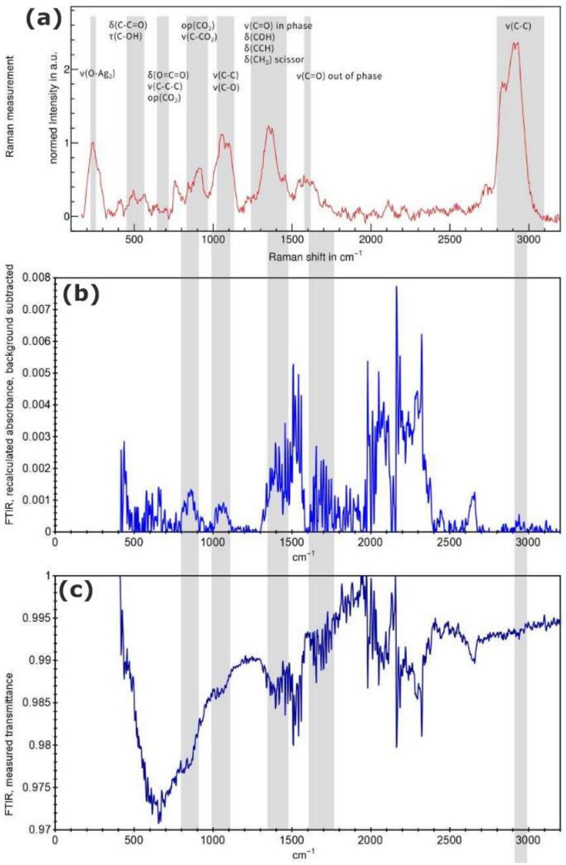

**Figure S14** Comparison of FTIR and Raman spectra showing in gray FTIR positions of functional bands of D-gluconic acid in literature.[1] (a) Raman spectra, (b) FTIR spectrum, background subtracted and converted to absorbance for comparison to Raman band positions achieved on Ag dendrites synthesized on Al foil in the presence of D-glucose in the solution, and (c) original FTIR spectrum.

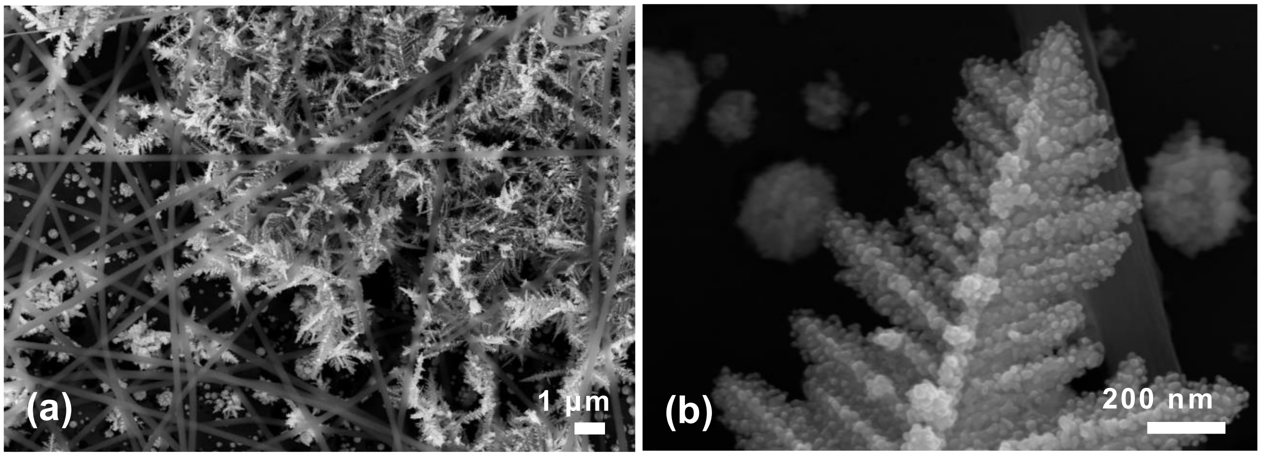


**Figure S15** SEM images (a) and (b) 3D hierarchical Ag nanodendritic structures formed on Al foil imaged after a period of 1 year.

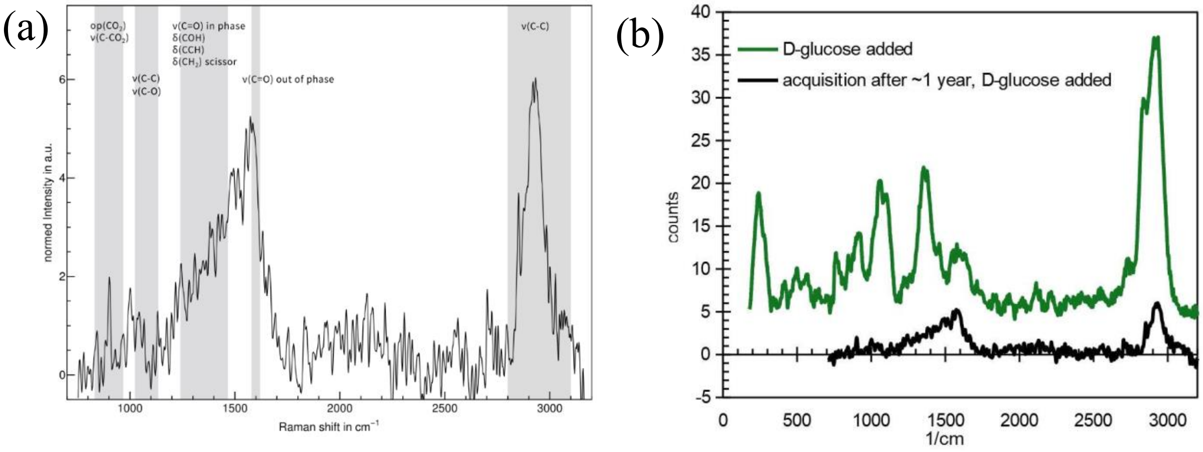


**Figure S16** (a) Averaged Raman spectrum of Ag dendrite positions acquired 1 year after sample fabrication by adding D-glucose during the production process; Single spectra are lowpass filtered before averaging; Highlighted vibrational modes of D-gluconate and D-gluconic acid are taken from literature[2,3] and indicate that D-gluconic acid is still present on dendrites. (b) Stability comparison of fresh vs. old sample. For a better visibility, the spectra were vertically shifted by 5 counts with respect to each other; the spectra count corresponds to a setting of 0.1 mW laser power and 1s acquisition time at a grating of 300 g/mm.

**References:**


[1] P.J. Linstrom and W.G. Mallard, Eds., NIST Chemistry WebBook, NIST Standard Reference Database Number 69, National Institute of Standards and Technology, Gaithersburg MD, 20899, https://doi.org/10.18434/T4D303,

[2] J. Kaminský, J. Kapitán, V. Baumruk, L. Bednárová, P. Bouř, *Journal of Physical Chemistry A* **2009**, *113*, 3594.

[3] I. O. Osorio-Román, V. Ortega-Vá Squez, C. Victor Vargas, R. F. Aroca, *http://dx.doi.org/10.1366/11-06279* **2011**, *65*, 838.
